# Myography of isolated blood vessels: Considerations for experimental design and combination with supplementary techniques

**DOI:** 10.3389/fphys.2023.1176748

**Published:** 2023-04-24

**Authors:** Rudolf Schubert, Dina Gaynullina, Anastasia Shvetsova, Olga S. Tarasova

**Affiliations:** ^1^ Physiology, Institute of Theoretical Medicine, Faculty of Medicine, University of Augsburg, Augsburg, Germany; ^2^ Faculty of Biology, M.V. Lomonosov Moscow State University, Moscow, Russia; ^3^ State Research Center of the Russian Federation, Institute of Biomedical Problems, Russian Academy of Sciences, Moscow, Russia

**Keywords:** myography, artery, smooth muscle, endothelium, membrane potential, intracellular calcium, innervation

## Abstract

The study of the mechanisms of regulation of vascular tone is an urgent task of modern science, since diseases of the cardiovascular system remain the main cause of reduction in the quality of life and mortality of the population. Myography (isometric and isobaric) of isolated blood vessels is one of the most physiologically relevant approaches to study the function of cells in the vessel wall. On the one hand, cell-cell interactions as well as mechanical stretch of the vessel wall remain preserved in myography studies, in contrast to studies on isolated cells, e.g., cell culture. On the other hand, *in vitro* studies in isolated vessels allow control of numerous parameters that are difficult to control *in vivo*. The aim of this review was to 1) discuss the specifics of experimental design and interpretation of data obtained by myography and 2) highlight the importance of the combined use of myography with various complementary techniques necessary for a deep understanding of vascular physiology.

## 1 Introduction

Diseases of the circulatory system like hypertension, diabetes and atherosclerosis are still accompanied by high morbidity and mortality. Therefore, the function of the cells of the vascular wall, i.e., endothelial cells, smooth muscle cells (SMCs) and also adipocytes as well as other cells of the adventitia is continuously in the focus of contemporary research. As far as the main function of the circulatory system, the distribution of blood according to local demand, is concerned, a variety of methods is employed to study the mechanisms governing the control of blood vessel tone.

Basic functions of the cells of the vascular wall can be addressed by applying a large array of molecular, biochemical, and physical methods on single cells, most often in cell culture. However, in cell culture cell-cell interactions inside the particular organ studied as well as interactions between different organs are lost. Moreover, cells in cell culture change gene expression quickly because they are kept in a growth promoting environment that has limited relevance for the physiological situation *in vivo*. SMCs, for example, alter their phenotype (conversion from the contractile to the synthetic phenotype). Thus, data obtained on single cells, in particular in cell culture, have to be verified in more physiological systems like intact isolated blood vessels or ultimately the intact circulatory system in order to extrapolate them to the *in vivo* situation.

Methods have been developed to study the contractility of isolated blood vessels from the largest vessel in the organism, the aorta, down to arterioles with a diameter of just a few micrometers. The basic principles of these methods (isometric and isobaric myography) have been described long ago (force measurement of large vessels (Furchgott and Bhadrakom, 1953), isometric tension measurement of small arterioles and arteries (Bevan and Osher, 1972; Mulvany and Halpern, 1976; Mulvany and Halpern, 1977; Mulvany et al., 1978), isobaric diameter measurement of smaller arteries (Halpern et al., 1984) as well as of very small arterioles (Duling et al., 1981; Vanbavel et al., 1990; Hoggerwerf et al., 1992; Coyne et al., 2002). Recently, important aspects of these methods have been addressed in a comprehensive review (Wenceslau et al., 2021) supplemented by two comments (Boedtkjer and Aalkjaer, 2022; De Mey et al., 2022), where the principles of isometric and isobaric myography and their application to vessels from different vascular beds have been summarized. Here we would like to complement this nice review i) by considerations regarding the design of experiments using these methods and ii) by describing their combination with methods to measure additional parameters, such as the expression level of signaling molecules, the membrane potential, the cytoplasmic calcium concentration and neuroeffector influences.

## 2 Myography: Initial distension and starting procedure

The first step in an experiment on isolated vessels is to set initial distension in order to ensure an optimal interaction of the contractile elements. This is a necessary procedure taking into account the length-dependence of the interaction of the contractile elements known for all types of muscles. Quite early this phenomenon was described also for large arteries (Dobrin, 1973) and then extended to smaller resistance vessels (Nilsson and Sjoblom, 1985).

The setting of initial tension is straightforward when isobaric vessel preparations are used. Here, just the pressure corresponding to the pressure experienced by the corresponding vessel *in vivo* is applied. The only limitation is that most often a constant pressure is employed, whereas *in vivo* pressure is pulsatile. Pulsatile pressure has its own effects (Speden and Warren, 1987; Loutzenhiser et al., 2002) (e.g., myogenic tone is determined by the systolic level of pulsatile pressure in renal small arteries (Loutzenhiser et al., 2002)). However, there is a lack of systematic studies examining the effects of pulsatile pressure on mechanisms that contribute to the regulation of vascular tone and have been previously explored with constant pressure. In addition, application of a physiological pressure to isobaric vessel preparations usually results also in longitudinal distension that has to be corrected for. Details on this issue have been published previously (Schubert, 2005).

For the setting of initial tension in studies using isometric vessels preparations, a recognized procedure exists, known also as “normalization”. Here, vessels are stretched until a tension is achieved that corresponds to a pressure slightly larger than 100 mmHg (13,3 kPa) as calculated by the law of Laplace. Based on the relationship between the internal circumference of the vessel and vessel tension, first the circumference of the vessel is determined, that corresponds to a pressure of 100 mmHg (IC_100_). Thereafter, tension is reduced by decreasing internal circumference by a certain degree, often by 10%, as at this decreased internal circumference optimal contractility has been observed (Mulvany and Halpern, 1977; Tarasova et al., 2003). Of note, the degree of initial tension set during normalization has to be established for different types of vessels. For example, porcine retinal arterioles with a diameter of about 150 μm are recommended to be stretched to an internal circumference of 0.9 *IC_70,_ where IC_70_ is the internal circumference at a transmural pressure of 70 mmHg (Hessellund et al., 2003). Further, pulmonary arteries should be stretched to IC_20_, taking into account the low arterial pressure in the pulmonary circulation (Shvetsova et al., 2022). A detailed description of the mathematical and biophysical basis for the normalization procedure is presented in previously published papers (Del Campo and Ferrer, 2015; Griffiths and Madhani, 2022).

Importantly, and probably often overlooked, the initial stretching procedure is aimed to determine the pure elastic properties of the vessel wall. Hence, the development of stretch-induced active tension should be avoided during this step. The development of active tension can easily be identified when after the stretch-induced passive increase and a subsequent initial decrease in tension, vessel tension starts to increase. However, often this is not so obvious and the existence of some active tension will be identified only when the initial stretching procedure is done in an external (bath) solution deprived of calcium ions (Hessellund et al., 2003; Shvetsova et al., 2022). Unfortunately, in most publications information on this topic is missing.

Last but not least, another commonly overlooked issue seems to be the fact that the reduction of internal circumference by as little as the often employed 10% means a reduction of vessel wall tension corresponding to a much reduced passive tension and transmural pressure (Schubert et al., 1996; Gaynullina et al., 2013). Table 1 shows examples of pressure values calculated according to Laplace’s law for different types of rat arteries stretched to an internal circumference equal to 0.9*IC_100_. Unfortunately, the transmural pressure in small arteries of awake animals has not been studied systematically. In small mesenteric arteries of awake rats a transmural pressure of about 65 mmHg has been determined (Fenger-Gron et al., 1995; Golubinskaya et al., 1999; Christensen and Mulvany, 2001). Thus, the values shown in table 1 seem to be close albeit a bit smaller than the *in vivo* transmural pressure.

**TABLE 1:** The characteristics of different rat arteries stretched to an internal circumference of 0.9*IC_100_: values of inner diameter, passive tension and corresponding levels of transmural pressure calculated according to Laplace’s law.

Type of artery	Inner diameter (μm)	Passive tension (mN/mm)	Transmural pressure (mmHg)
Mesenteric (7)	367 ± 63	1.11 ± 0.24	50 ± 5
Saphenous (7)	600 ± 49	1.88 ± 0.16	52 ± 3
Sural (8)	272 ± 30	0.90 ± 0.15	55 ± 9
Interlobar renal (6)	412 ± 54	1.18 ± 0.22	48 ± 5
Septal coronary (8)	290 ± 34	0.71 ± 0.11	41 ± 8
Basilar (7)	358 ± 35	0.82 ± 0.14	38 ± 6

The numbers in parentheses are the number of animals in the group. Data are presented as mean ± SD.

A discussion of some additional issues (e.g., ring preparations studied under isotonic conditions, role of the material of the mounting wires) has been published previously (Schubert, 2005).

Initial distension.

• isobaric vessel preparations: apply physiological pressure and correct longitudinal distension
• isometric vessel preparation: determine purely passive distension at which optimal contractility is achieved; calculate transmural pressure corresponding to this distension

Experiments on isolated vessels, whether isobaric or isometric preparations, often begin with a so-called starting procedure. During this procedure the vessel is challenged repetitively by contractile agonists after the potentially damaging vessel isolation and mounting procedure until a stable contractile response is achieved. In addition, a test to prove the existence/absence of a functional endothelium should be performed. Furthermore, in isometric preparations the starting procedure frequently includes the determination of maximum active tension as contractile responses often are normalized to this value.

Importantly, knowledge of maximum active tension is required when contractility has to be determined in absolute terms (for example, as vessel tension in mN/mm; tension = force/2*segment length). This may be required for the comparison of the contractility of, e.g., vessels from different vascular beds or of vessels from wild-type subjects and subjects deficient for certain molecules suggested to be important for vessel contractility. However, maximum active tension is heavily influenced by the vessel isolation and mounting procedures that are potentially damaging. Thus, whenever differences in maximum active tension are observed, this can be due to either real differences in the structure of the vascular wall (e.g., hypertrophy), or differences in contractile mechanisms on the cellular level, or damage induced during the vessel isolation and mounting procedure. The latter effect should be excluded by performing a number of control experiments in order to get evidence for a biological cause of possible differences in maximum active tension. For example, observing a thinner vascular wall will be consistent with a smaller maximum active tension and will provide evidence against the hypothesis that damage was the cause of the smaller active tension. Vessel wall thickness (better media thickness) can be estimated histologically or, which is easier, by light microscopy in mounted vessel segments (Mulvany et al., 1978; Puzdrova et al., 2014). In addition, the influence of vessel damage on experimental results should be reduced by random assignment of isolated vessels to different experimental groups and by an extensive training of the people mounting the vessels.

Starting procedure.

• recover contractile responses until sufficient and stable reactions are obtained; determine maximum active tension (cave: maximum active tension is sensitive to vessel damage)
• test the existence of sufficient reactions upon endothelial stimulation or their absence in endothelium-denuded preparations

## 3 Myography: Design of experimental protocols

Often, contractile responses of vessels, whether isobaric or isometric preparations, exposed to two or more conditions are compared. For example, vessels may be studied in the absence/presence of an ion channel or protein kinase inhibitor. Here, appropriate control experiments have to be performed and reported. Unfortunately, at least the latter is not done routinely.

Two protocols are principally possible. In the first, the sequential design, a vessel will be exposed to different conditions sequentially, e.g., the application of a contractile agonist initially in the absence and later in the presence of an ion channel blocker. Using this protocol, the contribution of the targeted ion channel to the effect of the contractile agonist can be studied. Of note, when more than one intervention is desired to be performed in the sequential design (e.g., different concentrations of the ion channel blocker), appropriate control experiments should verify that the first intervention does neither facilitate nor impede (e.g., due to incomplete washout or tachyphylaxis) the second intervention. For example, the effect of the BK channel blocker iberiotoxin is not fully reversible, thus any further intervention will be performed with at least partially blocked BK channels.

**FIGURE 1:**
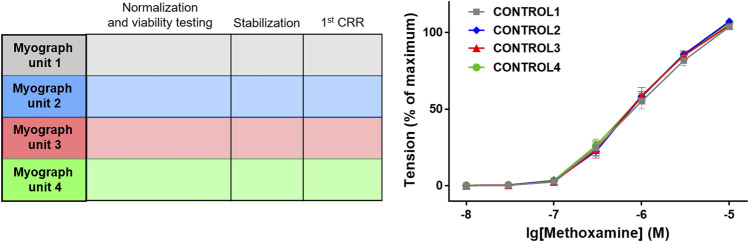
Control for similarity of initial contractility. As an example, the protocol of an experiment is shown in which 4 groups of blood vessels will be studied in myograph units 1–4. After the initial distension (i.e., normalization) and the starting procedure (i.e., viability testing) the effect of the contractile agonist methoxamine is tested by determining its concentration-response relationship (CRR) (protocol scheme shown in the left panel). As shown in the right panel, vessel tension in the 4 different experimental groups (CONTROL1—CONTROL4) were not different (*n* = 7; *p* = 0.94 based on the area under the concentration-response relationships). Reproduced and modified with permission from (Schmid et al., 2018).

More importantly, it should be considered that contractile responses of vessels, whether isobaric or isometric preparations, are not stable over time, as was shortly mentioned in (Wenceslau et al., 2021). Therefore, time-control experiments must be carried out. Optimally this is done in parallel, i.e., under the same conditions on a second vessel in the same or in a neighboring myograph. This represents the second design principle, the parallel design. For example, a contractile agonist was applied to one vessel initially in the absence and later in the presence of an ion channel blocker. In the appropriate time control the contractile agonist is applied twice, both times in the absence of the ion channel blocker with exactly the same timing of the application protocol and by adding the solvent of the ion channel blocker instead of the blocker itself. Importantly, the second (control) vessel should have an initial contractility similar to the contractility of the blocker-treated vessel. Thus, the response to the first addition of the contractile agonist studied should be similar in the control and the intervention group(s) (Figure 1). The latter is also true for relaxing agonists.

**FIGURE 2:**
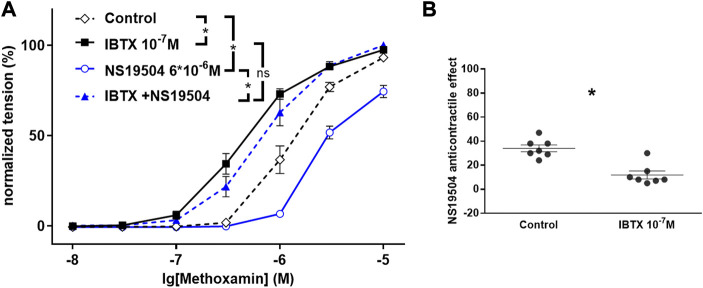
Effect of NS19504 and IBTX on methoxamine-induced contractions of the rat A. saphena. **(A)** Normalized tension (normalized to the maximum response of the 1st CRR-see Figure 1) of A. Saphena at different methoxamine concentrations in the absence of BK channel active agents (Control), in the presence of IBTX (IBTX 10^−7^ M), in the presence of NS19504 (NS19504 6*10^−6^ M) and in the combined presence of NS19504 and IBTX (NS19504 and IBTX). **(B)** NS19504 anti-contractile effect in the absence (Control) and presence of IBTX (IBTX 10^−7^ M). N = 7; * - *p* < 0.05. Reproduced and modified with permission from (Ma et al., 2020).

Of note, care should be taken when interpreting the results of complex experiments. For example, in the experiment shown in Figure 2 it was observed that the BK channel opener NS19504 alone had an effect when compared with the application of its solvent (control). In addition, it was shown that in the presence of IBTX no effect was detected for NS19504 when compared with the effect of IBTX alone. The finding that for NS19504 an effect was found in the absence of IBTX but was not detected in the presence of IBTX does not provide evidence that the effect of NS19504 is different in the absence and in the presence of IBTX. If it is desired to compare the sizes of the effect of NS19504 under both conditions, these effects should be quantified (e.g., as shift in pD_2_ or Δ area under the curve) and the resulting values be compared (see Figure 2B).

Design of experimental protocols.

• sequential design: time control experiments necessary; more than one intervention—test for complete reversibility of previous interventions
• parallel design: ensure similar initial contractility of parallel tested vessels

## 4 Myography: Analysis and interpretation of experimental data

### 4.1 Study of the mechanism of action of contractile or relaxing agonists in isobaric vessel preparations possessing pressure-induced tone

In mechanistic studies addressing the action of contractile or relaxing agonists, inhibitors (or activators) of potentially involved signaling pathways are often used. Notably, these inhibitors may affect contractility even before the addition of the contractile agonist because of a basal activity of the signaling pathway studied.

In isobaric vessel preparations possessing a myogenic tone at a physiologically relevant transmural pressure such basal activity is easily detected by a change in vessel diameter upon addition of the inhibitor. If such an effect is observed, an altered effect of the tested agonist in the presence of the inhibitor may be caused non-specifically due to the alteration of basal vessel tone induced by the inhibitor. Hence, appropriate controls have to be included to prove that the inhibitor specifically affects the action of the tested agonist.

**FIGURE 3:**
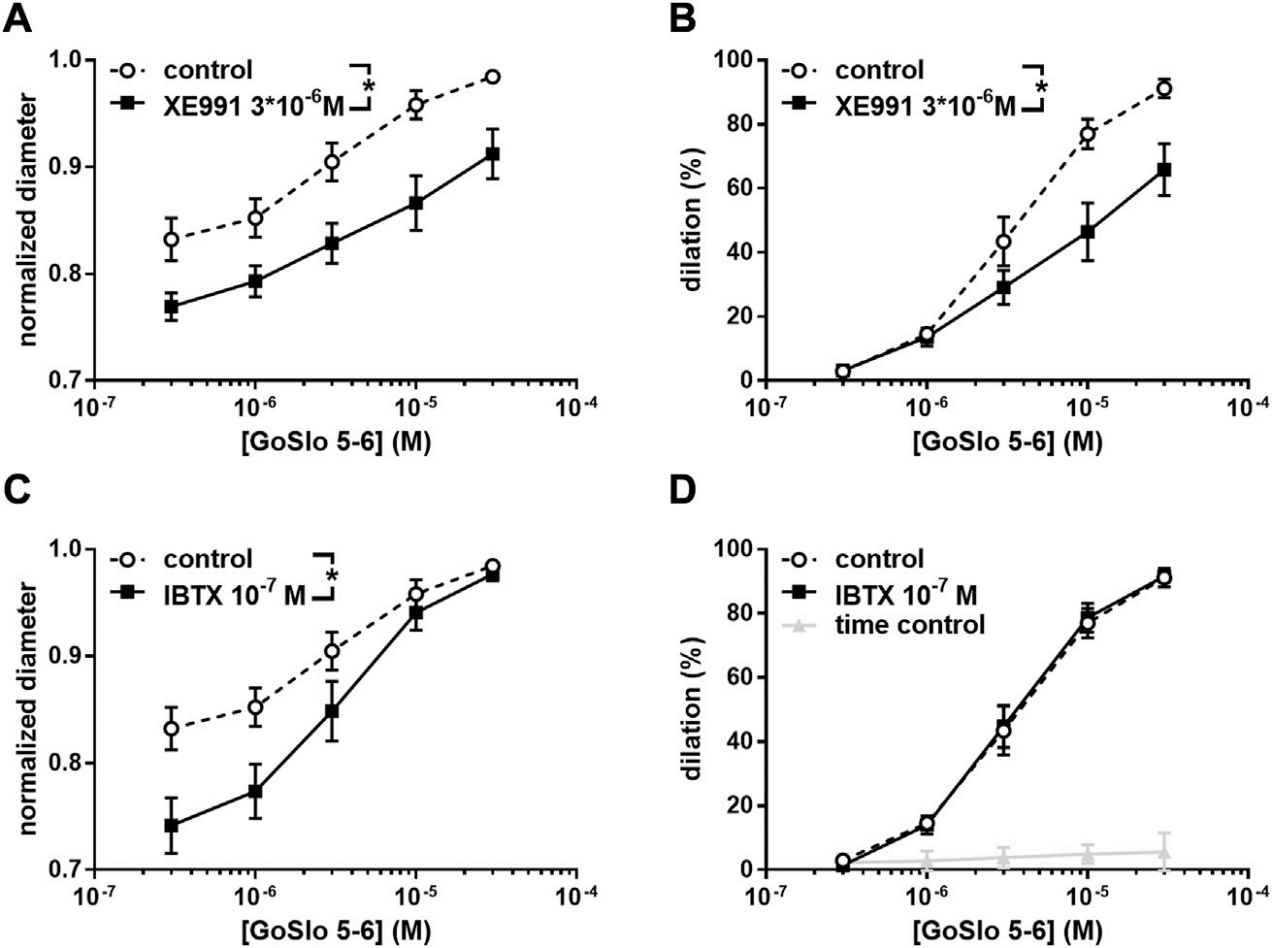
Effect of GoSlo-SR 5-6 on spontaneous tone of isobaric vessel preparations of rat Gracilis muscle arteries at 80 mmHg. **(A)** Normalised vessel diameter (ratio of diameter/fully relaxed diameter at 80 mmHg) at different concentrations of GoSlo-SR 5-6 in the absence (control) and presence of 3*10^–6^ M XE991 (*n* = 10; *p* < 0.05); **(B)** vessel dilation at different concentrations of GoSlo-SR 5-6 in the absence (control) and presence of 3*10^–6^ M XE991 (*n* = 10; *p* < 0.05); **(C)** Normalised vessel diameter at different concentrations of GoSlo-SR 5-6 in the absence (control) and presence of 10^–7^ M IBTX (*n* = 7; *p* < 0.05); **(D)** vessel dilation in the absence (time control = application of GoSlo-SR 5-6 solvent DMSO) and presence of GoSlo-SR 5–6 (control) (*n* = 7; *p* < 0.05), vessel dilation at different concentrations of GoSlo-SR 5-6 in the absence (control) and presence of 10^–7^ M IBTX (*n* = 7; *p* = 0.94). Reproduced and modified with permission from (Zavaritskaya et al., 2020).

Such control experiments may include, for example, the demonstration that another inhibitor targeting a synergistic signaling pathway (e.g., blocking another ion channel or protein kinase) does not change the effect of the tested agonist. For example, Figure 3 shows the dilating effect of GoSlo-SR 5-6 in rat small Gracilis muscle arteries. XE991, an inhibitor of Kv7 channels, reduced the effect of GoSlo-SR 5-6 partly (Figures 3A,B) but also decreased vessel diameter prior to GoSlo-SR 5-6 application (Figure 3A). Despite a similar change in vessel diameter induced by iberiotoxin (IBTX), an inhibitor of another K channel, the BK channel (Figure 3C), the effect of GoSlo-SR 5-6 was not found to be affected by IBTX (Figure 3D). Thus, the altered vessel diameter seems not to be the cause for the reduced dilating effect of GoSlo-SR 5-6 in the presence of XE991 (Zavaritskaya et al., 2020).

Alternatively, it can be shown that the effect of another agonist targeting a similar, parallel signaling pathway (e.g., affecting another, synergistic ion channel or protein kinase) compared to the initially studied agonist is not modified by the tested inhibitor.

In addition, the cited study (Zavaritskaya et al., 2020) contains a functionally interesting result that also points to the need for appropriate control experiments. Thus, the dilating effect of GoSlo-SR 5-6 was abolished in the combined presence of XE991 and IBTX. This finding suggests that GoSlo-SR 5-6 affected the vessels by activating both BK and Kv7 channels, with BK channels mediating the effect of GoSlo-SR 5-6 when Kv7 channels were unavailable. When Kv7 channels were available, they masked the contribution of BK channels to the effect of GoSlo-SR 5–6 (for more details see (Zavaritskaya et al., 2020)). Consequently, the contribution of BK channels would not have been discovered without the experiments addressing Kv7 channels. Therefore, in cases where synergistic signaling pathways exist (in this case, another potassium channel), it should be tested whether it masks the effect of the originally tested pathway or not.

These considerations in no way imply that all control experiments discussed have to be performed. Depending on the specific experimental conditions, the appropriate control experiments should be selected.

Analysis and interpretation of experimental myography data, preparations with spontaneous tone.

• consider possible effects of inhibitors/activators on basal activity of signaling pathways and perform appropriate control experiments
• consider masking effects of synergistic signaling pathways

### 4.2 Study of the mechanism of action of contractile or relaxing agonists in isometric vessel preparations and isobaric preparations without pressure-induced tone

The basal activity of a tested signaling pathway as discussed in the previous paragraph is more difficult to detect in isometric vessel preparations, particularly when the inhibitor has a relaxing effect and is applied to the vessel without agonist- or stretch-induced basal tone. Of note, this is true also for instances where larger vessels or some rare smaller arteries not able to develop pressure-induced tone are studied under isobaric conditions. In these cases, it should be considered that contraction has a certain threshold, i.e., that a minimum concentration of an agonist has to be applied to evoke contraction above passive basal tone. Of note, it has been observed that, e.g., membrane potential often has a lower threshold (see also the “Combining wire myography with membrane potential measurements” section); the same may be true for the intracellular calcium concentration. This means that these parameters may increase already at lower contractile agonist concentrations compared to vessel tension indicating that certain signaling pathways are active below the threshold for tone development, i.e., at basal tone. For example, if an inhibitor of a calcium signaling pathway is given to a vessel not possessing basal tone, tone will not change (decrease). If the subsequently added contractile agonist is observed to have a decreased effect, this is usually interpreted as a suppressive effect of the inhibitor on a calcium signaling pathway activated by the agonist assuming that the agonist increases the intracellular calcium concentration. However, at basal tone the inhibitor may have decreased the intracellular calcium concentration if the latter has a lower threshold than active tone. Let’s suppose that the contractile agonist in fact does not increase the intracellular calcium concentration but strengthens calcium sensitivity. Of note, pathways affecting calcium sensitivity and pathways altering the intracellular calcium concentration often interact. Thus, the inhibitor mentioned above, by producing a decrease of the intracellular calcium concentration, may have reduced the activity of the calcium sensitivity pathway as well. Consequently, the effect of the agonist acting through the calcium sensitivity pathway will be reduced. Thus, without measuring the intracellular calcium concentration, the reduced effect of the agonist can be misinterpreted as described above to indicate that the agonist increases the intracellular calcium concentration. Only having measured the intracellular calcium concentration would provide the correct interpretation that the effect of the agonist is mediated by a calcium sensitivity pathway. Consequently, whenever possible the measurement of additional parameters along with vessel tone or vessel diameter should be considered. Otherwise, the interpretation of the data should be done with great caution considering alternative explanations.

Analysis and interpretation of experimental myography data, preparations without spontaneous tone.

• consider possible effects of inhibitors/activators on basal activity of signaling pathways masked because they are occurring below the threshold of active tone development and perform appropriate control experiments by measuring additional parameters
• consider masking effects of synergistic signaling pathways

**FIGURE 4:**
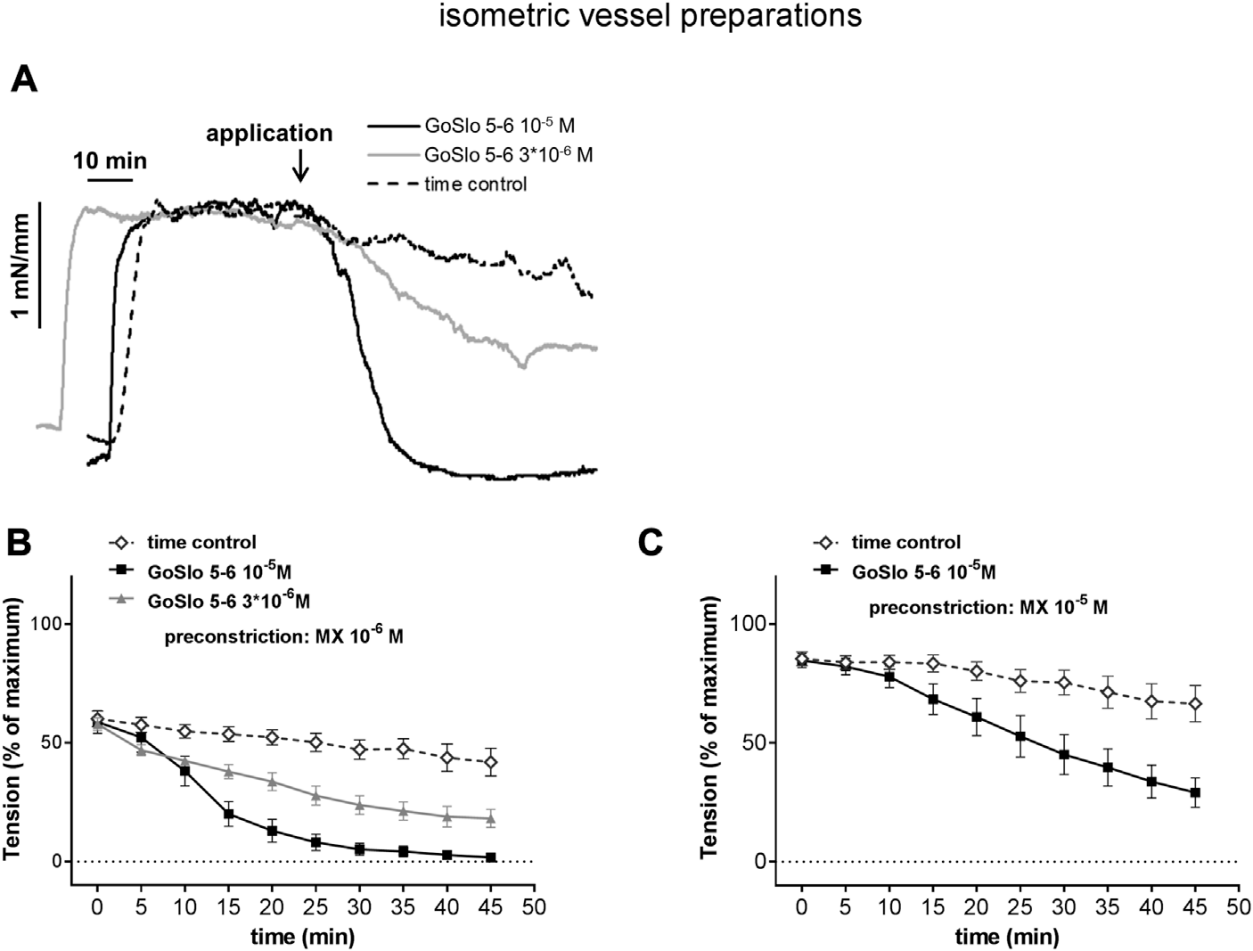
GoSlo-SR-5-6 causes concentration dependent relaxations of isometric preparations of rat Gracilis muscle arteries. **(A)** Example of the effect of GoSlo 5-6 on tension of an isometric vessel preparation at 10^–6^ M MX-induced tone. Application denotes the time point where GoSlo or vehicle was added. **(B)** Effect of GoSlo 5-6 on 10^–6^ M MX-induced contraction. Vessel tension in the absence (time control) and presence of GoSlo 5-6 at 3 × 10^−6^ M and at 10^–5^ M (repeated measures ANOVA: con vs. GoSlo 10^–5^ M - *n* = 11; *p* < 0.05; con vs. GoSlo 3 × 10^−6^ M—*n* = 8; *p* < 0.05; GoSlo 10^–5^ M vs. 3 × 10^−6^ M - *p* < 0.05). **(C)** Effect of GoSlo 5-6 on 10^–5^ M MX-induced contraction. Vessel tension in the absence (time control) and presence of GoSlo 5-6 at 10^–5^ M (repeated measures ANOVA: *n* = 8; *p* < 0.05). Reproduced and modified with permission from (Zavaritskaya et al., 2020).

When working with isometric vessel preparations or with isobaric preparations without pressure-induced tone, there is a special issue in experiments addressing the mechanisms of action of relaxing agonists. Here, a certain level of contraction, the so-called pre-contraction, has to be established by a contractile agonist. The effect of a relaxing agonist is often characterized by constructing concentration-response relationships. Since the application of a sufficient number of concentrations for a full concentration-response relationship requires quite some time, the pre-contraction has to be rather stable. However, often this is not the case (see Figure 4), challenging the unambiguous identification of weak relaxing effects. To demonstrate such effects, a time-control group should be included into the experimental design where the solvent of the agonist tested should be added in the same sequence as the agonist applying the same volume as during agonist application.

Further, there is a much more critical issue when studying the action of relaxing agonists. It is widely used practice to take the pre-contraction level as the baseline for calculating the degree of relaxation, i.e., the pre-contraction level is defined as 0% relaxation whereas a reduction of tension down to basal, passive tone is defined as 100% relaxation. This way of presenting data completely neglects the fact that the pre-contraction level may strongly affect the degree of relaxation obtained. For example, in Figure 4, where in deviation from common practice pre-contraction is expressed in percent of maximum tension, the substance GoSlo-SR 5-6 at 10^–5^ M produced full relaxation when applied at a moderate pre-contraction level (panel b) but only about 50% relaxation when applied at a somewhat stronger pre-contraction level (panel c). Let’s suppose, for example, that such a stronger pre-contraction was the result of the addition of an inhibitor of a certain signaling pathway. A smaller relaxing response in the presence of the inhibitor could be interpreted to indicate the involvement of the signaling pathway in the action of the relaxing agonist. However, in reality this difference was just the result of a functional antagonism, where the relaxing agonist had to “work” against much stronger contractile pathways. This misinterpretation can easily be avoided. There are two options. If the relaxing response is calculated using the pre-contraction level as baseline, the pre-contraction level itself should be reported as percent of maximum contraction. When two (or more) experimental groups will be studied, the respective pre-contraction levels should be shown to be equal (ideally using an equivalence test). A more comprehensive way to present data of the effect of relaxing agonists is to calculate all effects in percent of maximum contraction (together with the pre-contraction level) (see, for example, Figure 4; time point “0” represents the pre-contraction level) as this includes information about the pre-contraction levels in different data sets.

Regarding experiments addressing the mechanisms of action of relaxing agonists in isometric vessel preparations or in isobaric preparations without pressure-induced tone, an additional critical point appears when an inhibitor to be tested changes the level of pre-contraction. This is a similar situation as described above in the section “Study of the mechanism of action of contractile or relaxing agonists in isobaric vessel preparations possessing pressure-induced tone” and the same considerations for control experiments apply. In contrast to experiments on isobaric preparations with pressure-induced tone, there is an additional option. Here, the concentration of the contractile agonist employed to induce pre-contraction can be adjusted to ensure equal pre-contraction levels in the groups with and without the inhibitor.

Analysis and interpretation of experimental myography data, study of the mechanism of action of relaxing agonists after pre-contraction in preparations without spontaneous tone.

• in the case of weak relaxing effects perform time control experiments applying the solvent of the agonist tested
• report pre-contraction levels as percent of maximum contraction and show that they are equal; alternatively present all data in percent of maximum contraction
• if an inhibitor to be tested changes the level of pre-contraction, adjusting the concentration of the contractile agonist to induce pre-contraction can be considered to ensure equal pre-contraction levels in the groups with and without the inhibitor

**FIGURE 5:**
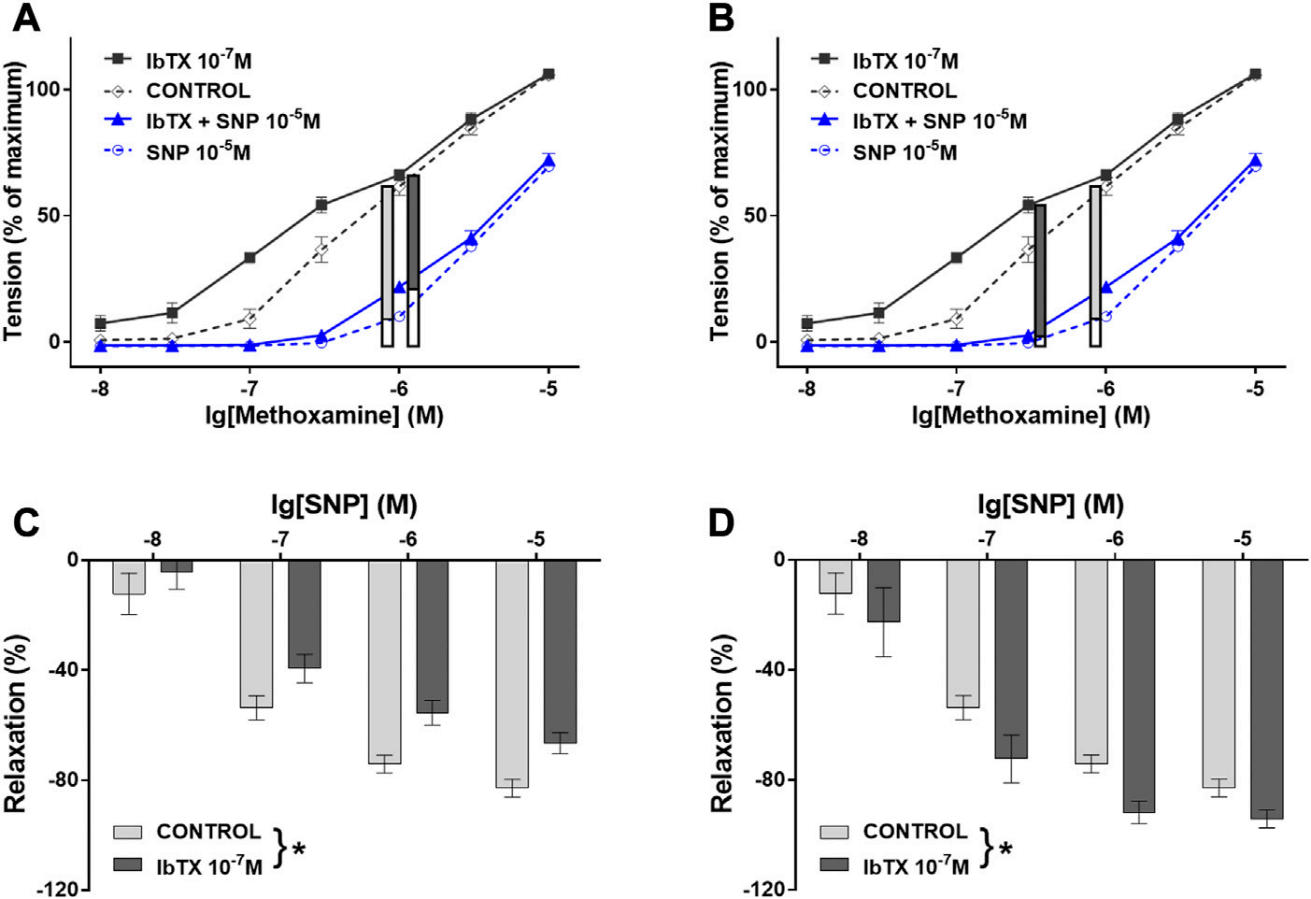
Contribution of the BK channel to the anti-contractile effect of SNP depends on the conditions used for pre-contraction. **(A)** and **(B)** Vessel tension in the absence of IBTX and SNP (CONTROL), in the presence of IBTX alone (IBTX), in the presence of SNP alone (SNP), and in the combined presence of IBTX and SNP (IBTX + SNP) at 10^–5^ M SNP. **(A)** Selection of similar levels of submaximal pre-contraction in the absence and presence of IBTX when IBTX had almost no contractile effect (black curves, 10^–6^ M MX); relaxing effect of 10^–5^ M SNP (blue curves) (filled parts of the vertical bars) in the absence and presence of IBTX presented also in **(C)** together with the effect of other concentrations of SNP (* - two-way ANOVA control vs. IBTX: *p* < 0.05); **(B)** Selection of similar levels of submaximal pre-contraction in the absence and presence of IBTX when IBTX had a considerable contractile effect (black curves, 3*10^–7^ M and 10^–6^ M MX); relaxing effect of 10^–5^ M SNP (blue curves) (filled parts of the vertical bars) in the absence and presence of IBTX presented also in **(D)** together with the effect of other concentrations of SNP (* - two-way ANOVA control vs. IBTX: *p* < 0.05); Reproduced and modified with permission from (Schmid et al., 2018).

The requirement to pre-contract vessels before the effect of relaxing agonists can be studied bears another challenge. In practice, the vessel is contracted to 50%–70% of maximum as this level of pre-contraction is more stable over time compared to lower levels of pre-contraction. However, depending on the conditions used for pre-contraction different pathways mediating vessel relaxation may dominate. Thus, a recent study addressed the contribution of BK channels to SNP-induced vessel relaxation (Schmid et al., 2018). To get comprehensive insight, a wide range of vessel contractility was studied by exploring the effect of SNP on an extensive concentration-response relationship for the contractile agonist methoxamine (MX). First, these data were analyzed mimicking widely used experimental designs, i.e., as if one vessel was exposed to a certain concentration of the contractile agonist (here 10^–6^ M MX) without the BK channel inhibitor iberiotoxin (IBTX) and a second vessel was exposed to the same concentration of MX in the presence of IBTX. Both pre-contraction levels were quite similar (see black and grey symbols at MX 10^–6^ M, Figure 5A). Analysis of the effect of SNP at a concentration of 10^–5^ M (Figure 5A, blue symbols at the same concentration of MX, filled parts of the vertical bars) and at several other concentrations (Figure 5C) showed that IBTX reduced the effect of SNP. This indicates that SNP can activate the BK channel, a finding consistent with a large number of previous studies. However, a different result was obtained when these data were analyzed as if one vessel was exposed to the same concentration of the contractile agonist as described above (here 10^–6^ M MX) without the BK channel inhibitor IBTX but the second vessel was exposed to a smaller concentration of MX in the presence of IBTX. Because at this smaller MX concentration IBTX had a considerable contractile effect, both pre-contraction levels were also quite similar (see grey symbol at MX 10^–6^ M and black symbol at MX 3*10^–7^ M, Figure 5B). Analysis of the effect of SNP at a concentration of 10^–5^ M (Figure 5B, blue symbols at both concentrations of MX, filled parts of the vertical bars) and at several other concentrations (Figure 5D) showed that IBTX increased the effect of SNP. This finding indicates that SNP can deactivate the BK channel.

**FIGURE 6:**
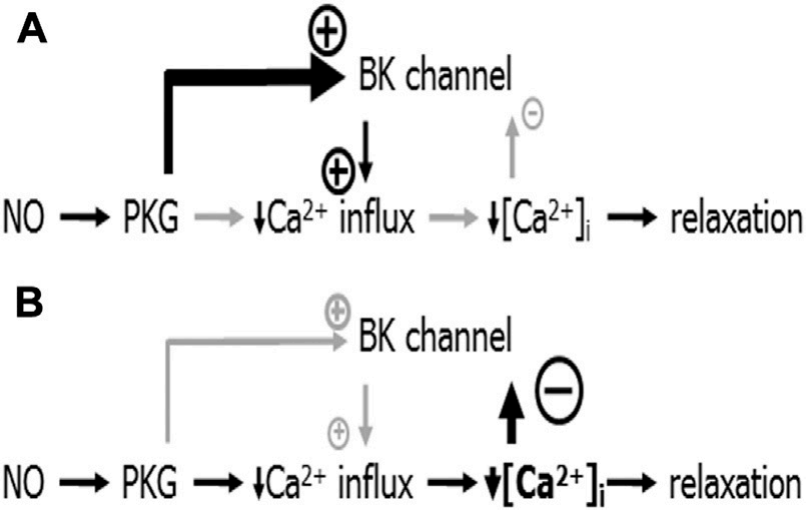
Schematic representation of the mechanism of BK channel contribution to the anti-contractile effect of SNP. **(A)** Conditions when NO produced only a small decrease in [Ca^2+^]_i_: NO induces a PKG-mediated activation of the BK channel that is stronger than the small [Ca^2+^]_i_-decrease-mediated deactivation of the BK channel. The overall effect of both is an activation of the BK channel contributing to the reduction of calcium influx; BK channels facilitate NO-induced vasodilation. **(B)** Conditions when NO produced a considerable decrease in [Ca^2+^]_i_: NO induces a PKG-mediated activation of the BK channel that is weaker than the large [Ca^2+^]_i_-decrease-mediated deactivation of the BK channel. The overall effect of both is a deactivation of the BK channel opposing the reduction of calcium influx; BK channels limit NO-induced vasodilation. Reproduced and modified with permission from (Schmid et al., 2018).

These findings have been explained based on the additional observation in the cited study that SNP produced a much smaller decrease in the intracellular calcium concentration (a key stimulus for BK channel activation) at higher concentrations of MX compared to its effect at smaller concentrations of MX (Schmid et al., 2018). It was suggested that at higher concentrations of MX SNP activates the BK channel via PKG to a larger degree than it deactivates the BK channel via the relatively small decrease in the intracellular calcium concentration (Figure 6A). Here, the BK channel promotes SNP-induced vessel relaxation. In contrast, at lower concentrations of MX SNP strongly decreases the intracellular calcium concentration and, therefore, deactivates the BK channel to a larger degree than it activates it via PKG (Figure 6B). Here, the BK channel can restrict SNP-induced vessel relaxations. Independent of the particular mechanism suggested, this example shows that the mechanism(s) of vessel relaxation should be studied under a variety of conditions, in particular at different conditions of pre-contraction.

The finding that different pathways of vessel relaxation may dominate depending on the conditions used for pre-contraction should also be considered when comparing data from isometric and isobaric vessel preparations. As discussed in the previous comprehensive review (Wenceslau et al., 2021), pre-contraction mechanisms differ because pressure (isobaric preparations) and agonists (isometric preparation) often use different signaling pathways. But even if the same pre-contraction pathways are activated in isobaric and isometric preparations, the functional consequences may differ. Thus, noradrenaline-induced depolarization was smaller in isobaric compared to isometric vessel preparations (Schubert et al., 1996). Since during contraction wall tension decreases in isobaric and increases in isometric preparations, changes in wall tension seem to contribute to the alteration in membrane potential induced by contractile agonists. As an additional example, it should be mentioned that the mechanisms of endothelium-dependent relaxation may differ under isobaric and isometric conditions. Thus, NO and a particular EDHF contributed to acetylcholine-induced relaxation in isometric preparations, whereas only EDHF, but another one, contributed to this response in isobaric preparations of mouse A. Gracilis (Boettcher and De Wit, 2011).

Analysis and interpretation of experimental myography data, study of the mechanism of action of relaxing agonists after pre-contraction in preparations without spontaneous tone.

• test different conditions of pre-contraction as they may affect the dominating pathway mediating vessel relaxation

## 5 Combining small vessel myography with mRNA and protein expression analyses

The investigation of the mechanisms of arterial contractile/vasodilator responses may require the use of additional methods targeted at, for example, the determination of the content of proteins or mRNA of interest in the vascular tissue. Additionally, it might be of interest to determine the degree of activation of the corresponding protein based on the level of its phosphorylation using phospho-specific antibodies. While the determination of the relative content of proteins or mRNA in arterial tissue does not raise questions (after isolation, the segment of the artery must simply be fixed in the appropriate buffer or frozen in liquid nitrogen), the determination of the content of the phosphorylated protein often requires its preceding activation using vasoactive substances. This section will describe how a wire myograph can be used for the preparation of arterial tissue for further analysis by Western blotting using phospho-specific antibodies, as well as the preparation of endothelium-denuded arterial tissue for this purpose. Of note, a commercially produced pressure myograph for rapid freezing or fixing of intact, pressurized vessels is available. Its special chamber is made of special inert material to withstand low temperatures and fixatives.

### 5.1 Determining protein phosphorylation/signaling pathway activity

In some cases, it may be desired to determine protein phosphorylation levels under certain conditions, for example, following the action of a vasoconstrictor or a specific inhibitor. Since continuous measurements of protein phosphorylation, similar to vessel tension or vessel diameter measurements, are not possible, the level of protein phosphorylation should be determined at those moments that correspond to the most relevant time points according to the experimental protocol (Lubomirov et al., 2006; Gaynullina et al., 2013; Gaynullina et al., 2018; Mochalov et al., 2018). In such cases, it is possible, and may be even recommended, to use an adapted wire myograph for sample preparation. In this procedure, the mounted vessel segments are subjected to the same starting and treatment procedures as during usual functional experiments on the myograph. At a time point of interest, the preparation is quickly removed from the myograph wires and fixed (e.g., with a 15% trichloroacetic acid/acetone/dry ice slurry) for further protein isolation and Western blotting using phospho-specific antibodies (Lubomirov et al., 2006; Gaynullina et al., 2013; Gaynullina et al., 2018; Mochalov et al., 2018; Selivanova et al., 2021).

**FIGURE 7:**
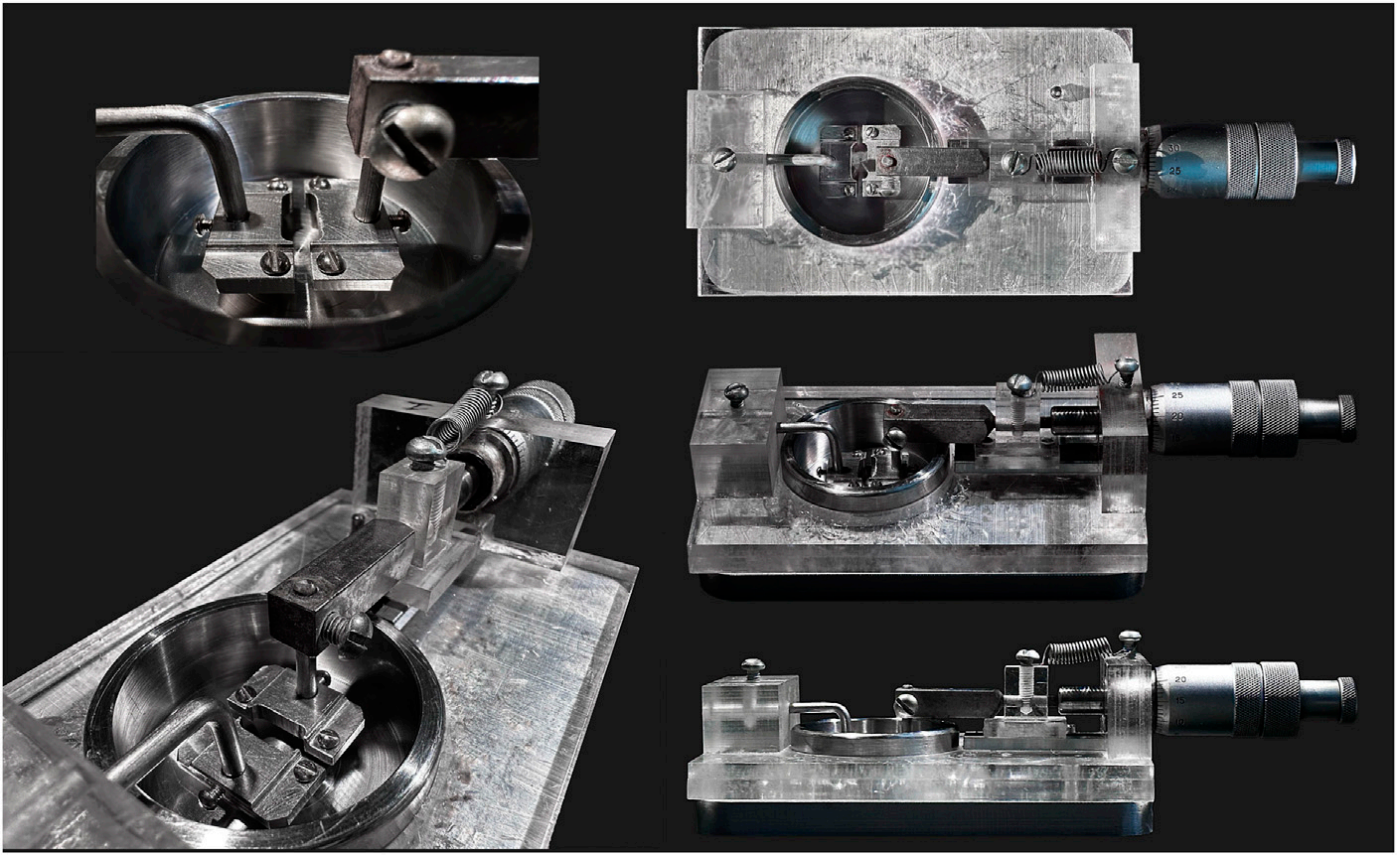
Custom-made wire myography analogue. This device is equipped with wider heads to increase arterial segment length up to 10 mm. It has a micrometer that allows to change the distance between the heads but has no force transducer.

Of note, when using a standard wire myograph, the length of an arterial segment in the myograph chamber will be 2 mm at maximum. In some cases this is not enough to obtain a sufficient amount of protein. Thus, either several preparations must be combined into one sample. Alternatively, to increase the length of arterial segments, we developed a custom analogue of the myograph with wider heads to increase segment length up to 10 mm (Figure 7). This wire myograph analogue is equipped with a micrometer that allows the distance between the heads to be changed to stretch the preparation but does not have a force transducer. Due to the absence of a force transducer, the standard normalization procedure cannot be performed. However, stretching of the preparation may be based on data previously obtained in a real wire myograph or obtained in parallel experiments in a real myograph for the studied type of artery from age- and weight-matched animals. One of the advantages of using such a device is that its upper part, together with the heads on which the preparation is mounted, is removable, so that one can quickly transfer it into a 15% trichloroacetic acid/acetone/dry ice slurry without detaching the arterial preparation from the wires. In this case, the very rapid fixation of the preparation allows the study of the content of phosphoproteins that usually rapidly dephosphorylate, for example, phospho-MLC20.

### 5.2 Endothelium-denuded sample preparation

In some cases, it is desirable to carry out PCR and/or Western blotting on vessels without endothelium. This is necessary, for example, to determine the localization of certain genes and/or proteins in SMCs or endothelial cells. One possible approach here is the mechanical removal of the endothelium in vascular ring segments using the wire myograph system, followed by rapid tissue processing (Shvetsova et al., 2019; Shvetsova et al., 2020; Kostyunina et al., 2020). To do this, the vessels should be isolated in a cold physiological buffer on ice. Thereafter, the vessels should be transferred to the myograph chamber and mounted on wires. The myograph chamber should also be pre-cooled and filled with cold solution (+4°C). Removal of the endothelium is carried out using a rat’s whisker or human hair. Then the vessel is cut longitudinally and washed to remove endothelial cell debris. Subsequently, vessel preparations can be frozen in liquid nitrogen (for later isolation of RNA or proteins), fixed in RNA-later (for later isolation of RNA) or in a trichloroacetic acid/acetone/dry ice slurry (for later isolation of proteins). Of note, all manipulations should be carried out as fast as possible in order to prevent the degradation of RNA and proteins.

The success of endothelial removal can be controlled by the level of mRNA or protein expression specific for endothelial cells. To do this, arterial samples with endothelium should be prepared as reference samples using the same procedure as described above but without removing the endothelium. In this case, the amount of vascular tissue in samples with and without endothelium should be comparable. During PCR or Western blotting, the expression levels of mRNAs or proteins specific for endothelial cells (e.g., eNOS, vWF, *etc.*) should be determined simultaneously in endothelium-intact and endothelium-denuded samples. The expression level of mRNAs or proteins (after normalization to a reference gene or protein, which should have the same expression level in SMCs and endothelial cells) should be several times lower in samples without endothelium than in samples with endothelium (Shvetsova et al., 2019; Shvetsova et al., 2020; Kostyunina et al., 2020).

Combining small vessel myography with mRNA and protein expression analyses.

• consider the use of a special myograph to get larger amounts of protein from small vessels
• in case of denudation of the endothelium, mRNA or protein expression specific for endothelial cells (eNOS, vWF, *etc.*) should be compared in endothelium-denuded and endothelium-intact samples in order to confirm the success of endothelium removal

## 6 Combining small vessel myography with membrane potential measurements

Vascular tone is largely determined by the membrane potential of SMCs (Nelson et al., 1990). Thus, the simultaneous recording of arterial contraction and membrane potential allows to explore the mechanisms of vascular tone control associated, in particular, with changes in the functional state of ion channels in the plasma membrane. The recording of the membrane potential of vascular SMCs using the sharp microelectrode technique has several special features due to the considerably smaller size and higher input resistance of SMCs compared, for example, to cardiomyocytes or skeletal muscle cells.

A microelectrode, usually made of aluminosilicate or borosilicate glass, must have a very thin tip (no more than 1 µm in diameter) in order to penetrate a small cell without damaging it. In this regard, microelectrodes in electrophysiological studies of arterial smooth muscle have a relatively high resistance - at least 30 MΩ, e.g., (Wu et al., 2004; Boedtkjer et al., 2008; Dam et al., 2014; Shvetsova et al., 2019; Shvetsova et al., 2020). Importantly, high-resistance electrodes are often characterized by a large tip potential that has been shown to reduce the recorded membrane potential values (Adrian, 1956; Purves, 1981). Therefore, the tip potential of the microelectrodes used should be evaluated. Noteworthy, the tip potential is reduced when freshly prepared microelectrodes with microfilaments filled with 3 M KCl are used (Okada and Inouye, 1975; Plamondon et al., 1976; Gagne and Plamondon, 1983).

Recording of the membrane potential should be carried out on an antivibration table to prevent the possible escape of the microelectrode from the impaled cell in case of any mechanical disturbances. The position of the microelectrode in relation to the studied artery should be controlled in two stages. In the first stage of “rough” adjustment, the microelectrode is manually moved using a manipulator macro-screw to a position where the tip of the electrode is close to the vessel but does not touch it yet. In the next stage, just before and during the impalement, a more subtle way of changing the position of the microelectrode is required. For this purpose, for example, a Piezo micromanipulator should be used, which allows to move the electrode over several micrometers.

**FIGURE 8:**
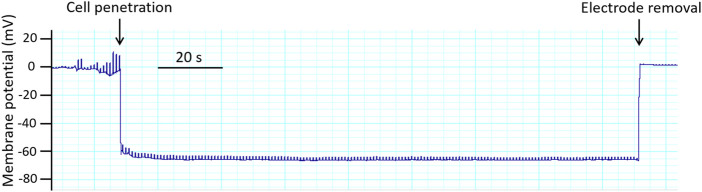
Original trace obtained in an experiment with membrane potential registration in rat saphenous artery. Membrane potential recordings are accepted according to the following criteria: (i) a sharp drop of the potential on cell penetration; (ii) a stable level of the membrane potential recording for at least 30 s; (iii) a return to the zero-potential level after electrode removal; and (iv) a similar electrode resistance before and after the measurement (Nilsson et al., 1998; Rummery and Brock, 2011; Shvetsova et al., 2019).

The thin and long tip of the microelectrode can easily be clogged with fragments of connective tissue or bent on contact with the vessel wall. This will lead to the appearance of artifacts (signal fluctuations), which the experimenter may mistake for the impaling of a cell. Therefore, it is important to control the input resistance of the microelectrode tip throughout the experiment by applying subthreshold (short and low-amplitude) current pulses (may be except of periods used to record example traces for illustration). A sharp increase in the amplitude of these pulses will indicate that the tip of the microelectrode rests against the vessel wall, is bent, or is contaminated. When the tip of the microelectrode has penetrated the cell successfully, the amplitude of the responses to the current pulses should be small since the total resistance of the microelectrode tip and the smooth muscle syncytium is low (Figure 8). Special acoustic systems (for example, Audis-01D/16, npi electronic, Germany) can be used as an additional control for the position and condition of the microelectrode tip. Each pulse applied through the tip of the microelectrode is converted into a tone and is heard as a high-pitched sound. When the tip of the microelectrode is bent or contaminated, a specific noise appears. Upon successful entry of the microelectrode into the cell, a “clear” high-pitched sound would be heard. Of note, to reduce the possibility of damage and/or contamination of the microelectrode most vessels should be cleaned of connective tissue very carefully before mounting the vessel in the myograph.

It is well known that depolarization of SMCs leads to contraction, and hyperpolarization, on the contrary, to relaxation of arteries (Nelson et al., 1990; Thorneloe and Nelson, 2005; Tykocki et al., 2017). However, the dependence of contraction on membrane potential in arterial SMCs is non-linear. In other words, during stimulation arterial smooth muscle does not begin to contract until a certain threshold membrane potential is reached. Thus, in experiments on rat small mesenteric and tail arteries an increase of the extracellular potassium concentration up to 20–25 mM induced depolarization of SMCs by 10–15 mV which was not associated with the development of a contractile response (Mulvany et al., 1982; Neild and Kotecha, 1987). In our previous experiments on saphenous arteries of adult rats the blockade of Kv7, Kir2 or TASK-1 channels (by XE991 (3 µM), BaCl_2_ (30 µM) or AVE1231 (1 µM), respectively) depolarized arterial smooth muscle by about 10 mV, while it did not induce the development of a contractile reaction (Shvetsova et al., 2019; Shvetsova et al., 2020). Therefore, the absence of an effect of a substance on arterial tone does not exclude the possibility of an effect on the membrane potential, which is important to consider when interpreting such experimental data.

**FIGURE 9:**
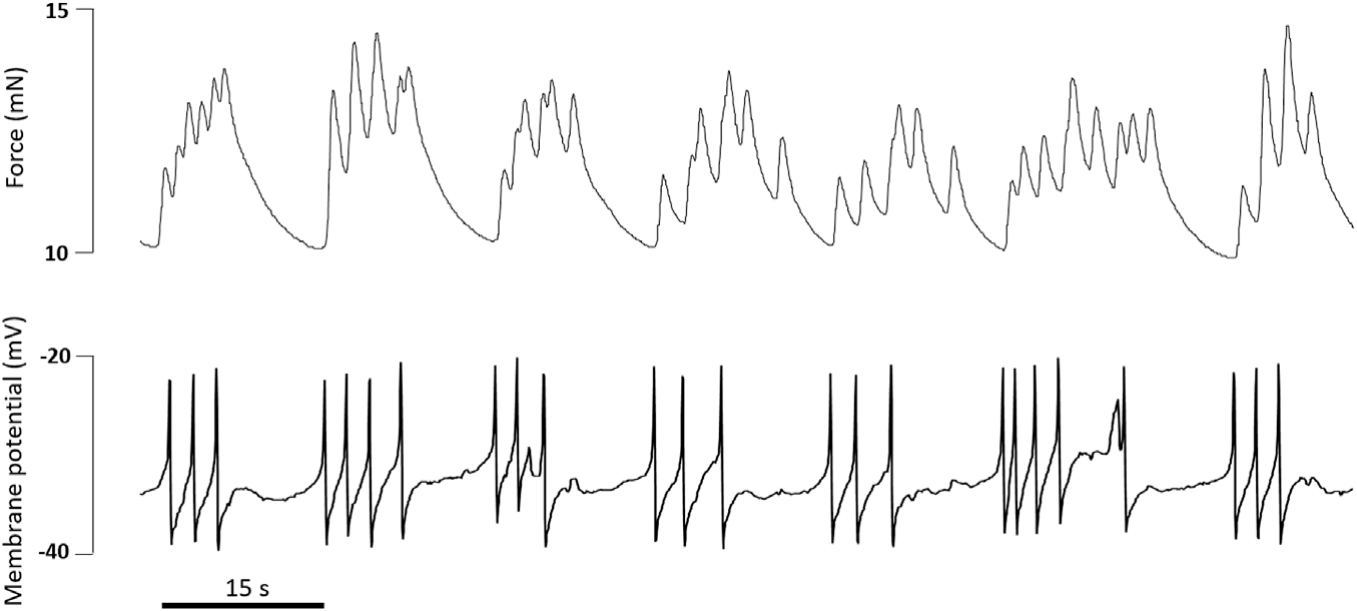
Original traces of force and membrane potential simultaneously recorded in an experiment on rat tail artery. Incubation with the α_1_-adrenoceptor agonist methoxamine (0.4 µM) and the BK_Ca_ channel blocker Iberiotoxin (0.1 µM) caused the development of action potential-like spikes accompanied by phasic oscillations of tone.

The combined use of the myograph technique and membrane potential measurements contributed much to the understanding of the mechanisms of vasomotion—rhythmic contractions of small arteries and arterioles (Aalkjær and Nilsson, 2005; Cole et al., 2019). Vasomotion occurs in the presence of some contractile agonists and/or under the blockade of certain potassium channels and is associated with oscillations of membrane potential that precede the oscillations of force (Harder and Sperelakis, 1979; Gustafsson and Nilsson, 1994; Aalkjær and Nilsson, 2005). Moreover, potassium channel blockade can lead to the appearance of potential-like spikes, which is a non-typical electrical activity for arterial SMCs (“tonic” phenotype of smooth muscle) (Harder and Sperelakis, 1979; Itoh et al., 1981; Hirst et al., 1986) (Figure 9), pointing to an important role of potassium channels in the maintenance of a stable resting membrane potential.

Combining small vessel myography with membrane potential measurements.

• assess the microelectrode tip potential in order to obtain exact values of the membrane potential
• evaluate the input resistance of the microelectrode tip throughout the experiment to avoid the appearance of artifacts and to confirm successful impalement
• consider different thresholds contractile agonists may have for inducing changes in vessel tension and membrane potential

## 7 Combining small vessel myography with calcium fluorimetry

The mechanisms determining vascular tone may affect the intracellular calcium concentration as well as the calcium sensitivity of the contractile elements (see, e.g., (Somlyo and Somlyo, 2003) and also chapter “Study of the mechanism of action of contractile or relaxing agonists in isometric vessel preparations and isobaric preparations without pressure-induced tone” above). Because of this dual mode of vascular tone regulation, measurements of vessel tension or diameter alone will not provide unambiguous information as to whether a change in vascular tone is caused by an alteration of the intracellular calcium concentration or of the calcium sensitivity of the contractile elements. Thus, clarification of the contribution of changes of the intracellular calcium concentration to vascular tone regulation as well of its spatial organization requires simultaneous recording of vascular tone and of the intracellular calcium concentration.

The intracellular calcium concentration can be measured using either synthetic calcium dyes, or genetically encoded calcium indicators, or the newer calcium-responsive nanoparticles, or novel calcium synthetic biology approaches including optical control strategies (optogenetics) (Li and Saha, 2021). While the latter two methods are techniques in development, genetically encoded calcium indicators are more widely used. They are based on fluorescent proteins that are produced by translation of a nucleic acid sequence, in particular after their integration into the genome of transgenic organisms. Thus, limitations due to exogenous dye loading and problems with dye uptake into organelles, as observed with synthetic calcium dyes, are avoided. However, due to the longer time required for the translation process compared to the acute loading procedure of synthetic calcium dyes, together with the calcium buffering properties of all calcium sensing dyes, they can induce profound, long-term changes in the cells studied.

Most commonly, the intracellular calcium concentration in cells of the circulatory system is measured using synthetic calcium dyes. There exist a large range of such dyes from high affinity ones (dissociation constant K_d_ <1 μM, useful for the determination of the cytosolic calcium concentration) to low affinity ones (K_d_ > 1 μM, useful for the determination of the calcium concentration in different organelles). Detailed descriptions of the general methodological background of these methods can be found elsewhere, e.g., (Grynkiewicz et al., 1985; Takahashi et al., 1999; Li and Saha, 2021).

**FIGURE 10:**
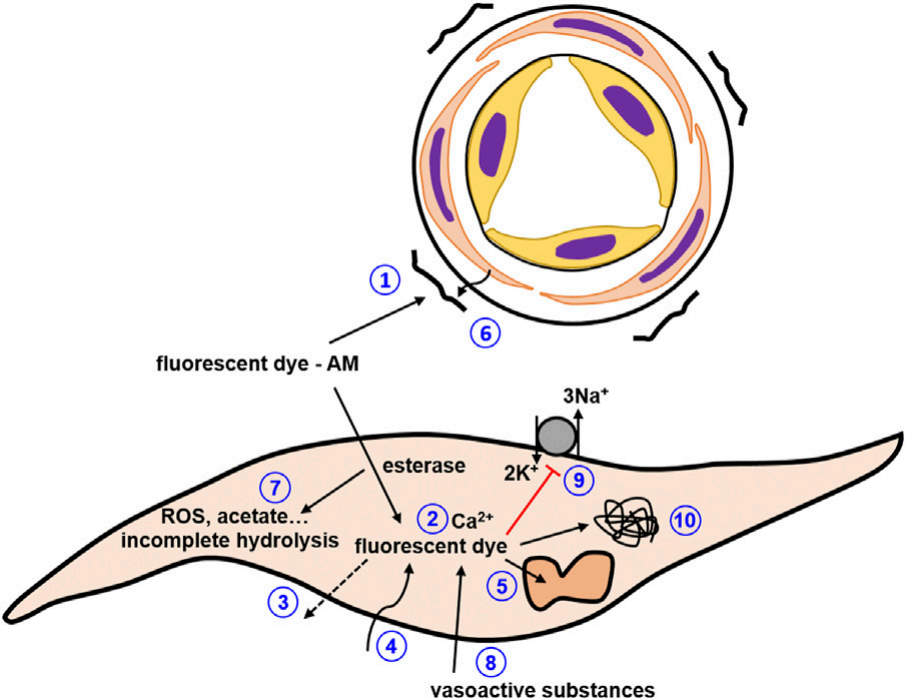
Pitfalls to be considered in studies with synthetic calcium dyes. **1)** dye precipitates stick to the adventitia adding a strong calcium-insensitive signal; **2)** buffering of intracellular calcium by calcium dye affecting the calcium concentration as well as the kinetics of calcium concentration changes; **3)** dye loss; **4**) photobleaching; **5)** compartmentalization of dyes; **6)** screening or scatter of fluorescence by connective tissue; **7)** phototoxicity and incomplete hydrolysis; **8)** vasoactive substances alter fluorescence of the dye or contribute to the fluorescence signal; **9)** inhibition of Na/K ATPase especially at high dye concentrations; **10)** interaction with intracellular proteins..

In a large number of studies exploring changes in the cytoplasmic calcium concentration in cells of the circulatory system, fluorescence has been collected from the entire vessel preparation, i.e., as an integrated signal from all cells of the vessel wall (either isobaric or isometric preparations). For this purpose, FURA-2 has often been used because it allows ratiometric measurements (Grynkiewicz et al., 1985; Jensen et al., 1992). This dye emits fluorescence both in its calcium-free and in its calcium-bound form, emitted light is collected at 512 nm upon sequential dual excitation at 340 and 380 nm. Ratiometric measurements strongly reduce artefacts induced by movements of the preparation, dye loss, photobleaching, *etc.* (Figure 10, #3/4). Of note, FURA-2 fluorescence is independent of the intracellular calcium concentration when excited at a special wavelength, the isosbestic point. Thus, recording fluorescence at this wavelength can provide information about measurement artefacts like, for example, movements of the preparation, dye loss, and photobleaching. Commonly, the isosbestic point for FURA-2 is observed at 360 nm. However, fluorescence spectra, including the isosbestic point, depend on the intracellular environment (Owen, 1991). Therefore, evaluating measurement artefacts based on FURA-2 fluorescence at the isosbestic point can have its own pitfalls. However, if the influence of an experimental intervention on the isosbestic point is unknown, FURA-2 spectra can be recorded at different calcium concentrations.

For the investigation of the spatial organization of changes in the intracellular calcium concentration confocal microscopy has been employed on vessel preparations. Using this method, changes in the intracellular calcium concentration can be detected in either single smooth muscle cells or single endothelial cells (Simpson, 1999; Paredes et al., 2008; McCarron et al., 2012; Dora and Hill, 2013; Wilson et al., 2015; Lawton et al., 2019) or even in both together when a special arrangement of the vessel is used (Rahman et al., 2007). However, since the dyes used in confocal microscopy are usually non-ratiometric, artefacts induced by movements of the preparation, dye loss, photobleaching, *etc.*, are rather critical. Especially movement artefacts have to be avoided. This can be achieved either by strongly immobilizing the vessel preparation, which is non-physiological, or by techniques to correct for motion artifacts (e.g., (Fenrich, 2020; Vogt et al., 2021)).

The loading of synthetic calcium dyes into the cells of isolated vessel preparations is quite straightforward. Commonly, these dyes are polycarboxylate anions and therefore are not able to cross the lipid bilayer. For better membrane permeability these dyes are usually modified to acetoxymethyl esters. They are dissolved in an appropriate solvent, most often DMSO, and the vessel preparation is incubated with the dye for an appropriate time. Dye loading can be facilitated using dispersing agents like Pluronic F-127 or Cremophor EL. In some cells this loading procedure can produce dye concentrations in the cytosol of hundreds of µM with just several µM in the loading medium (Oakes et al., 1988). This is important as higher dye concentrations in the loading medium exceed the solubility limit of the dye, leading to precipitation of the dye. Of note, such precipitates often stick to the adventitia of vessel preparations and add a strong calcium-insensitive signal to the measurement (Figure 10, #1). Upon entering the cells, intracellular esterases hydrolyze the dyes making them available for the detection of the intracellular calcium concentration. Of note, incomplete hydrolysis of fluorescent dyes has been observed (e.g., (Oakes et al., 1988; Roe et al., 1990)) that can contribute to background fluorescence. After loading, some time is required to remove the acetoxymethyl moiety. Therefore, optimal loading conditions should be established for the preparation under investigation that result in a sufficient increase in fluorescence after loading compared to background with minimal side effects.

Several additional critical points should be considered regarding the loading procedure. The acetoxymethyl moiety does not only facilitate dye crossing of the cytoplasmic membrane, but also the crossing of intracellular membranes. This leads to a compartmentalization of the dyes into intracellular organelles (Figure 10, #5). In addition, cells can endocytose these dyes into some of their organelles. Since the calcium concentration in these compartments is usually quite high, this will bias the measurement of the intracellular concentration towards higher values. Whereas loading of dye esters into compartments cannot be avoided, the endocytotic process can be reduced by loading at lower temperatures. Because the loading procedure itself is also temperature-dependent, as a compromise loading is often performed at room temperature. However, the conditions for optimal loading have to be established empirically. If it is suspected that compartmentalization may be a problem, a simple test can be employed. First, the cytoplasmic membrane is selectively permeabilized with digitonin, releasing the cytosolic dye. Then, the organelle membranes are permeabilized with Triton X-100, releasing the compartmentalized dye (see, for example, (Roe et al., 1990; Kao et al., 2010)). Based on these measurements it can be determined how much of the total intracellular dye is compartmentalized in organelles.

The ability to obtain fluorescence from dyes to estimate the intracellular calcium concentration depends not only on successful loading of the dye. Another critical point is the connective tissue surrounding all vessels to a larger or smaller extend. Sometimes this connective tissue is able to screen or scatter the fluorescence from successfully loaded dyes quite strongly (Figure 10, #6). In this case, simultaneous measurements of vessel tension and, e.g., FURA-2 fluorescence show an unaltered FURA-2 emission signal accompanied by changes in tension, suggesting a contractile mechanism that acts independently of changes in the intracellular calcium concentration. Such misinterpretation can be avoided by a very careful dissection of the connective tissue and obligatory control experiments showing that after dye loading appropriate stimuli, e.g., 120 mM KCl (solution prepared by equimolar replacement of NaCl), can induce changes in fluorescence.

Pitfalls to be considered in studies with synthetic calcium dyes.

• dye loss
• photobleaching
• dye precipitates stick to the adventitia adding a strong calcium-insensitive signal
• compartmentalization of dyes
• screening or scatter of fluorescence by connective tissue

When changes of the intracellular calcium concentration are assessed using fluorescent dyes, attempts are often made to express the signal in terms of concentrations. This can be done by either calibration of the fluorescence signal using predefined calcium calibration buffers or by calculations (see, for example, Grynkiewicz et al., 1985; Kao et al., 2010). The latter requires knowledge of the calcium dissociation constant K_d_ of the dye and of the fluorescence in the absence of calcium (F_min_) and the presence of saturating calcium (F_max_) for non-ratiometric dyes or of the fluorescence ratio in the absence of calcium (R_min_) and in the presence or saturating calcium (R_max_) for ratiometric dyes. For the measurement of the latter factors, an equilibrium should be established between the calcium concentration in the external bath solution and in the cytosol, usually by the addition of an ionophore such as ionomycin. For FURA-2 this is usually done in a 3-step procedure. 1) Incubation in bath solution containing ionomycin and EGTA to reduce the calcium concentration and to get R_min_; 2) Incubation in bath solution containing ionomycin and a high calcium concentration to get R_max_; and 3) addition of Mn^2+^ quenching FURA fluorescence to get background fluorescence (see, e.g., Jensen et al., 1992).

Although this seems to be a straightforward approach, it is not. Thus, especially for non-ratiometric dyes the determination of minimum and maximum fluorescence is often impossible due to non-avoidable dye leakage from the preparations and photobleaching of the dye during the course of an experiment. For ratiometric dyes, other limitations make the determination of these factors difficult. Because ionomycin is not very effective in an environment with a low calcium concentration, determination of R_min_ requires a long time and the true R_min_ may even not be reached. In contrast, during the determination of R_max_ in an environment with a high calcium concentration, ionomycin will facilitate cell lysis and dye loss from the preparation. In addition, the fluorescence at 380 nm obtained under these conditions, especially at the end of longer experiments, is often close to the background fluorescence at 380 nm, making the determination of the fluorescence ratio (R = F340/F380) difficult due to the division by a very small number.

Moreover, there is another issue common to all types of calcium dyes, the K_d_ should be known. However, the binding of calcium to a fluorescent dye is highly dependent on a number of conditions, including pH, ionic strength, the presence of other ions that also bind to the dye, and other substances that directly influence the dye. Since the intracellular values of many of these factors are not known, the K_d_ of a fluorescent dye determined outside the cell of interest *in vitro* is not a good estimate of the real K_d_ value (Jensen et al., 1993). Thus, if calibration in terms of calcium concentration is desired, K_d_ should be selected or determined with great care.

In addition, the calcium calibration buffers with free calcium concentrations in the physiological range required for the determination of K_d_ or for the direct calibration of the florescence signal deserve special consideration. The theory for the preparation of such solutions has long been known in detail (e.g., Schubert, 1996). Thus, the composition of these solutions can be calculated with a number of published programs. However, several practical problems in preparing such solutions exist. These include unknown purities of buffer substances, which in reality may differ from the manufacturer’s specifications (Tran et al., 2018), trace contaminations in the electrolyte components, *etc.* Therefore, such calculations can only provide an estimate of the solution composition, and the concentration of free calcium must be measured. However, the device used to measure the free calcium concentration must also be calibrated, a “Catch22”situation. In the recently described method (Tran et al., 2018) a calcium-sensitive macroelectrode was used that was calibrated with solutions containing higher calcium concentrations not requiring calcium buffers. However, the behavior of the electrode in the physiological range of calcium concentrations had to be extrapolated and showed a non-linear behavior depending on a number of conditions. Alternatively, commercially available calcium calibration solutions can be used. However, we have found that the concentrations of free calcium in these solutions vary by up to 1 pCa unit between batches from one manufacturer and between different manufacturers, making their use very unreliable. Taking into account the many uncertainties associated with the calibration of the fluorescence signal, it might be better to report only fluorescence (for non-ratiometric dyes) or ratio (for ratiometric dyes) values or to express the data as the percentage of the difference between R_max_ and R_min_ (Puzdrova et al., 2014).

**FIGURE 11:**
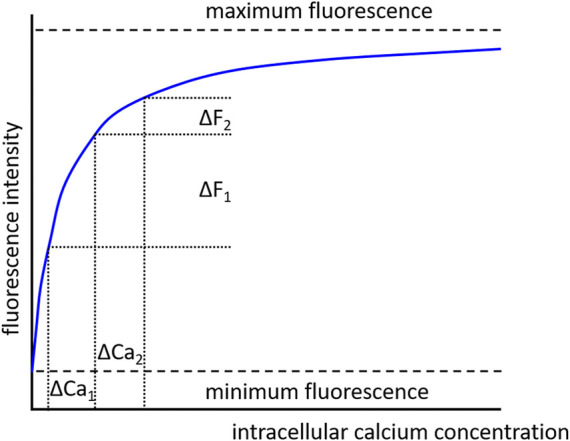
Dependence of fluorescence intensity on calcium concentration. In the absence of calcium, the dye is free of calcium and minimum fluorescence is observed. With increasing calcium concentration the dye binds increasingly more calcium ions, but when all dye molecules are occupied by calcium ions a maximum fluorescence is achieved. There is a non-linear dependence of fluorescence intensity on calcium concentration. Consequently, similar changes in calcium concentration (ΔCa1 = ΔCa2), when evoked from different initial calcium concentrations, produce different changes in fluorescence intensity (ΔF1 > ΔF2).

Of note, it should be considered that fluorescence intensity is a non-linear function of the calcium concentration. This means that similar changes in fluorescence do not represent similar changes in calcium concentration when the baseline values are different (see Figure 11). Therefore, it is recommended to use dyes with a K_d_ value appropriate for the expected calcium concentration so that saturation of the dye does not occur, and the measurement is made in the steeper part of the relationship between intracellular calcium concentration and fluorescence.

Another important point, regardless of whether a calibration is performed or not, is the consideration of background fluorescence. Especially when examining intact vessel preparations, the background fluorescence can be quite strong and must absolutely be subtracted from the fluorescence signal. Background fluorescence can be determined by excitation of the vessel preparation before loading. In this case, loading must be done on the microscope stage to maintain the selected field of view. Alternatively, it can be done at the end of the experiment after permeabilization with, e.g., ionomycin and quenching of the dye fluorescence with Mn^2+^. This seems to be a straightforward procedure. However, it has its limitations. Background fluorescence may change during longer experiments due to bleaching, and this loss can occur in a non-linear manner. Careful consideration should therefore be given to whether the background obtained is representative of the experiment as a whole. Information about background correction belongs obligatorily in the method section of papers.

There are several additional points to be taken into account. Thus, different substances used to evoke changes in vessel contractility can affect fluorescence independently of changes in the intracellular calcium concentration (Figure 10, #8). This may be because these substances themselves are excited and contribute to the fluorescence signal or because they alter the fluorescence properties of the dye (e.g., Kopp et al., 2014; Paudel et al., 2014; Santofimia-Castaño et al., 2016). As a first test, the interaction of the investigated substance with the free salt of the selected fluorescent dye can be studied *in vitro*. Several other limitations are based on the properties of the dyes themselves, as recently detailed, along with suggestions for control experiments (Roe et al., 1990; Bootman et al., 2018). Briefly, due to their chemical nature, calcium sensitive fluorescent dyes act as calcium buffers (Figure 10, #2). If this effect is larger than the effect of intracellular endogenous buffers, the dye will be the dominating buffer altering the dynamics of changes in the intracellular calcium concentration. Of note, this property of calcium sensitive fluorescent dyes can be used to study characteristics of intracellular calcium handling, e.g., the calcium buffering capacity (for more details see Takahashi et al., 1999). Further, some fluorescent dyes based on BAPTA have been shown to affect membrane transport processes, e.g., to inhibit the Na/K-ATPase and glucose uptake independently of changes of the intracellular calcium concentration, i.e., affect cellular function directly (Figure 10, #9). Moreover, it is suggested that a large amount of dye molecules bind to intracellular proteins, changing the fluorescence of the dye and possibly the functionality of the intracellular proteins (Figure 10, #10). In addition, fluorescent dyes can be phototoxic when excited, generating, for example, reactive oxygen species or acetate, which can impair cellular functions (Figure 10, #7). Since the limitations mentioned above are based on the properties of the dyes themselves, loading of fluorescent dyes should aim to obtain only the minimum intracellular concentration necessary for reliable measurement of the desired response.

Pitfalls to be considered in studies with synthetic calcium dyes.

• calibration of fluorescence signals in terms of calcium concentrations is associated with many uncertainties; reporting only fluorescence or ratio values should be considered
• background fluorescence should be subtracted and this procedure be reported
• vasoactive substances may alter fluorescence of the dye or contribute to the fluorescence signal
• buffering of intracellular calcium by calcium dyes affect the calcium concentration as well as the kinetics of calcium concentration changes
• inhibition of Na/K ATPase especially at high dye concentrations
• interaction with intracellular proteins
• phototoxicity and incomplete hydrolysis

## 8 Studying vascular neuroeffector mechanisms by small vessel myography

Studies *in vitro* serve to explore the presynaptic and postsynaptic mechanisms of vascular neurotransmission, because nerve fibers (the so-called “intramural nerves”) remain in the vessel wall after it is isolated. They stay functional (capable of storing and secreting neurotransmitters) for several hours, and with proper storage even for several days after vessel isolation from the body (Yang and Chiba, 1999b).

Three types of vasomotor nerve fibers can be found in blood vessels: sympathetic, sensory, and parasympathetic (Burnstock and Ralevic, 2014; Roloff et al., 2016; Xavier, 2020; Aalkjær et al., 2021). Sympathetic fibers operate with three main neurotransmitters: noradrenaline, adenosine triphosphate (ATP), and neuropeptide Y (NPY) that commonly induce SMC contraction through α-adrenoceptors (predominantly α_1_), P2X (predominantly P2X1) receptors and Y1 receptors, respectively. In addition, noradrenaline can induce vasorelaxation by activating β-adrenoceptors. Direct effects of NPY are hardly observed *in vitro*, but it can considerably potentiate the effects of noradrenaline and ATP (Prieto et al., 2000; Burnstock and Ralevic, 2014; Aalkjær et al., 2021; Gonzalez-Montelongo and Fountain, 2021).

The neurotransmitters used by vascular sensory nerves are calcitonin gene related peptide (CGRP), substance P, neurokinins, and, presumably, anandamide (Aalkjær et al., 2021). CGRP-ergic vasodilatory effects are most often observed upon the activation of sensory nerves in isolated arteries, the contribution of other proposed transmitters in neurogenic vasodilatation has not been commonly observed (Wiencke et al., 1994; Aalkjær et al., 2021). The parasympathetic vasodilatory neurotransmitters are acetylcholine, vasoactive intestinal peptide (VIP), and nitric monoxide (NO) (Roloff et al., 2016; Xavier, 2020). Presumably, NO can also originate from sensory nerves, there is no commonly accepted point of view on this matter yet (Aalkjær et al., 2021).

### 8.1 Electrical field stimulation of intramural nerves: Rules and pitfalls

To activate intramural nerves electrically, a myograph must be equipped with stimulating electrodes. The electrodes (usually made of platinum) are placed 1–3 mm from each side of the vessel without touching it. Thus, the nerves are excited by the electric field passing through the physiological salt solution (electrical field stimulation - EFS) (Nilsson, 1984; Sjöblom-Widfeldt, 1990). Stimulation is carried out by rectangular current pulses with a sufficiently high amplitude (up to 100 mA) that originate from a high-powered electrical stimulator (*e.g.,* CS100, Danish Myo Technology). The electrodes can be either wires (about 1-mm thick) (Nilsson, 1984; Sjöblom-Widfeldt, 1990) or plates (Anschütz and Schubert, 2005). In the second case, current density is a more correct indicator of stimulus strength (Anschütz and Schubert, 2005). It is better if the pulses are delivered with alternating polarity in order to avoid electrolysis in the salt solution around the electrodes.

#### 8.1.1 Distinguishing the effects of EFS on nerve fibers and smooth muscle cells

Importantly, current pulses can induce excitation not only of intramural nerves, but also of SMCs of the vessel wall. However, the threshold for stimulation of nerve fibers is lower than that of SMCs, so it is possible to define parameters at which the electric current selectively excites nerve fibers. These parameters may differ for preparations from different vessels. Therefore, when starting to work with a new type of vessels, it is necessary to prove the neurogenic nature of the response to electrical field stimulation. In most studies, this is done using tetrodotoxin (TTX), which blocks voltage-gated sodium (Na_v_) channels of nerve fibers and hence the neurogenic vasomotor responses (Nilsson, 1984; Sjöblom-Widfeldt, 1990).

It should be noted that TTX-sensitive Na_v_ channels, found in electrophysiological studies of vascular SMCs (Berra-Romani et al., 2005; Saleh et al., 2005), are not involved in the regulation of SMC contractile activity under normal conditions (Saleh et al., 2005; Ho et al., 2013). Apparently, this is due to the depolarized membrane potential of SMCs, at which most Na_v_ channels are inactivated (Berra-Romani et al., 2005; Saleh et al., 2005). In support of this, reversal of Na_v_ channel inactivation with veratridine was shown to cause vessel contraction (Saleh et al., 2005; Ho et al., 2013). In addition, the density of Na_v_ channels in SMCs may be lower than in nerve fibers, making nerve fibers more excitable by EFS.

Ideally, current strength-duration curves (i.e., the relationships between pulse duration and pulse amplitude for minimal detectable responses) should be plotted in the absence and in the presence of TTX for the studied vessel type (Nilsson, 1984). The parameters of the pulses at which selective stimulation of nerve fibers occurs are determined by the geometry of the electrodes and the distance between them and the preparation. For example, the stimulating electrodes can be placed at each end of the vessel segment, causing an electrical field in the longitudinal direction of the vessel. Alternatively, the electrodes can be placed at each side of the vessel segment to cause an electrical field in the transverse direction. Since during transverse stimulation the current runs along the circularly oriented SMCs, the input resistance and activation threshold of the SMCs are lower than during longitudinal stimulation, when the current runs across the cells. Therefore, during transverse stimulation the gap between threshold values for activating the nerves and SMCs directly is narrower, as compared to longitudinal stimulation. For the same pulse amplitude, the pulse duration can be up to 2 ms for longitudinal current, and should be limited to 0.1–0.2 ms for transverse orientation (Sjöblom-Widfeldt, 1990). However, transverse stimulation is the more common approach, especially in wire myograph studies, due to the convenience of mounting the electrodes in the myograph heads.

Notably, a subset of small dorsal root ganglion neurons was shown to express TTX-resistant Na_v_ channels (Akopian et al., 1996) that can participate in the propagation of the action potential along C-type sensory fibers (Brock et al., 1998). However, the role of TTX-resistant Na_v_ channels in the release of transmitters from nerve terminals *in vitro* has been investigated in only a few studies. In guinea pig atrial preparations, TTX-resistant inotropic responses were not observed during EFS, but were induced with the application of bradykinin (Geppetti et al., 1990). Along with that, in murine small mesenteric arteries mounted in a wire myograph EFS induced TTX-resistant relaxations, which could be blocked by the Na_v_ 1.8 channel antagonist A-803467 or by capsaicin (Miranda-Morales et al., 2010).

Therefore, in most cases, the effects of EFS on nerve fibers and SMCs can be proven using TTX. Possible neurogenic mechanisms of TTX-resistant responses to EFS can be identified with the use of capsaicin or antagonists of neurotransmitter receptors (for more detail see “Analysis of the effects of sensory/parasympathetic nerves” section).

#### 8.1.2 Potential endothelium-dependent effects of EFS

The endothelium can modulate neurogenic responses of isolated vessels through various mechanisms. Firstly, neurotransmitters released from intramural nerves may diffuse to the endothelium and activate it. For example, activation of the endothelium by neuronal acetylcholine was shown in guinea-pig basilar arteries (Jiang et al., 1997). Cholinergic endothelium-dependent modulation of the neurogenic relaxation was also observed in the rabbit middle cerebral artery (Van Riper and Bevan, 1992).

Next, the endothelium may be activated secondarily to the activation of SMCs. Such a mechanism of endothelium-dependent suppression of EFS-induced constrictor responses was demonstrated in isobarically pressurized third-order mouse mesenteric arteries (Nausch et al., 2012). The authors attributed this effect to IP3 diffusion through myoendothelial gap junctions that are abundant in smaller arterial vessels (Sandow and Hill, 2000).

Further, the results of several studies suggest a direct influence of the stimulation current on endothelial cells of small arteries (Buga and Ignarro, 1992; Sullivan and Davison, 2001; Kun et al., 2003; Anschütz and Schubert, 2005). Interestingly, the endothelium of human umbilical artery and vein can synthesize dopamine and release it in response to EFS (Britto-Júnior et al., 2020). However, the mechanisms of endothelium activation by EFS were not explored in these studies. Several studies reported the presence of Na_v_ channels in vascular endothelial cells (Figueroa et al., 2007; Lillo et al., 2021; Hernández-Plata et al., 2023) but their activation upon EFS is rather questionable due to the fairly depolarized resting membrane potential of endothelial cells (Siegl et al., 2005; Stankevičius et al., 2006). However, even without voltage-gated channel activation, EFS may change the potential on the endothelial cell membrane electrotonically and, therefore, the driving force for ion currents across it. Regardless of the mechanism involved, a potential role of the endothelium in modulating the responses to EFS should not be forgotten.

#### 8.1.3 Strong EFS can induce non-neurogenic vasorelaxation

Notably, EFS may be accompanied by phenomena that are not related to the direct effect of the current on vascular cells. In the study by Hardebo et al. EFS of arterial or venous segments from different mammals induced a relaxation that persisted in the presence of TTX or capsaicin, as well as after endothelium denudation and omission of extracellular calcium (Hardebo et al., 1989). At moderate intensities of EFS a TTX-resistant response was presumably due to the electrolytic formation and action of chlorine gas whose bubbles were observed near the electrodes. At strong stimulation intensity TTX-resistant relaxation was prevented by ascorbic acid, reduced glutathione, catalase or superoxide dismutase pointing to the formation of reactive oxygen species during EFS.

Electrolytic formation of vasorelaxant molecules during high-intensity EFS was also reported in several studies published later. TTX-resistant relaxation in response to EFS was observed in pre-contracted rat basilar artery segments; the response was not affected by a large number of channel/receptor blockers and endothelium removal, but was inhibited by superoxide dismutase (Conde et al., 1999). Similarly, in the study by Varma et al. (Varma et al., 2006) EFS induced TTX-resistant and endothelium independent relaxation in a wide spectrum of vascular preparations (rat aorta and portal vein, rabbit aorta and renal artery, dog coronary artery, pig ductus arteriosus). The relaxation was also observed during exposure of the preparations to “electrically stimulated” physiological salt solution. The substance produced by EFS that triggers the relaxation was identified by the authors as sodium hypochlorite, a strong oxidant (Varma et al., 2006).

Therefore, when performing EFS in experiments on isolated arteries, it is extremely important to ensure the neurogenic origin of the response. Notably, with correctly selected parameters, the scavenging of free radicals did not affect EFS-induced vessel responses (Anschütz and Schubert, 2005). Since non-neurogenic reactions usually manifest as vasorelaxation (Hardebo et al., 1989; Conde et al., 1999; Varma et al., 2006), preparations not possessing a spontaneous tone should be pre-contracted pharmacologically (with noradrenaline, vasopressin, *etc.*) before testing the sensitivity of EFS-induced reactions to TTX, capsaicin or other appropriate pharmacological tools that will be discussed in the next section.

Electrical field stimulation of intramural nerves:

• in most cases, the neurogenic response is blocked by TTX
• the effects of sensory nerves containing TTX-insensitive Na_v_ channels can be eliminated by capsaicin
• vascular endothelium can modulate vascular responses to stimulation of sympathetic, sensory and parasympathetic nerves
• high-current stimulation can induce vasorelaxation due to the formation of chlorine gas or reactive oxygen species potentially damaging to vascular wall cells

### 8.2 Pharmacological analysis of nerve fiber types and their postjunctional effects

The next task when studying neuroeffector mechanism is to understand what type of nerve fibers is activated upon EFS and what neurotransmitter induced the vasomotor response. This can be done using pharmacological tools that disrupt i) the secretion of transmitters from nerve fibers or ii) the interaction of neurotransmitters with their receptors (receptor antagonists). In the first case, it is important to verify the selective presynaptic action of secretion disruptors (the absence of their effects on vascular smooth muscle). In the second case, it is necessary to prove the blockade by stimulating the receptors (with the transmitter itself or its analogue specific for a certain receptor subtype) against the background of antagonist action. The agonist should be applied at a submaximal concentration, or at least at a concentration that causes a vasomotor effect comparable in amplitude to the neurogenic response. Care should also be taken in choosing the concentration of the antagonist because at high concentrations antagonists may have undesirable side effects.

#### 8.2.1 Analysis of the effects of sympathetic nerves

For identifying the gross effects of sympathetic nerve fibers, the pharmacological tools of choice are 6-hydroxydopamine (6-OHDA) and guanethidine. Incubation of isolated vessels with 6-OHDA provides *in vitro* sympathectomy (Aprigliano and Hermsmeyer, 1976). An undoubted advantage of this approach is the ability to perform experiments after washout of the drug from the vessel. However, this experimental protocol requires a relatively long time (more than an hour) and the use of a modified physiological salt solution (to prevent oxidation of 6-OHDA at physiological pH). An alternative approach could be to use 6-OHDA *in vivo* by injecting the drug into the animal a few days prior to the experiment (Ralevic and Burnstock, 1996). 6-OHDA accumulates in sympathetic varicosities by an uptake-1 transport mechanism and then generates highly reactive oxygen species that destroy the neuronal structures (Kostrzewa and Jacobowitz, 1974). Importantly, 6-OHDA induces destruction of adrenergic nerve terminals without direct effects on SMCs (Aprigliano and Hermsmeyer, 1976). However, it should be considered that the noradrenaline-depleting effect of 6-OHDA is less pronounced and recovers more rapidly in vascular than in other tissues (Finch et al., 1973).

Guanethidine blocks adrenergic transmission by two mechanisms that are also associated with its uptake by sympathetic fibers (Brodie et al., 1965). First, it prominently depletes noradrenaline stores in sympathetic nerve fibers when injected into the animal 4–6 h before tissue isolation (Cass and Sproggs, 1961). Second, within several minutes after application, guanethidine was shown to disrupt excitation-secretion coupling mechanisms in sympathetic varicosities, presumably, by altering the properties of the synaptic vesicle membrane (Brock and Cunnane, 1988). In addition, when applied at higher concentrations, guanethidine blocks impulse conduction in sympathetic nerve terminals (local anesthetic-like effect) (Brock and Cunnane, 1988). Besides noradrenaline, guanethidine inhibits EFS-induced secretion of NPY (Donoso et al., 2004) and ATP (Yang and Chiba, 1999a), although the purinergic component of the neurogenic response may be less sensitive to guanethidine than the adrenergic one (Yang and Chiba, 1999a).

The adrenergic component of the sympathetic response can be dissected out using the pan-α_1_-adrenoceptor antagonist prazosin (Sjöblom-Widfeldt, 1990; Sjoblom-Widfeldt et al., 1990) or subtype-specific tools, such as RS100329 for α_1A_ and BMY7378 for α_1D_ receptors (Mittal et al., 2020). To produce a combined blockade of α_1_-and α_2_-adrenoceptors, the appropriate tools could be phentolamine (Nilsson, 1984; Anschütz and Schubert, 2005; Mittal et al., 2020) or the receptor-alkylating agent phenoxybenzamine (Tarasova et al., 2003). Notably, α_2_-adrenoceptor antagonists, such as idazoxan, yohimbine or rauwolscine, can induce relaxation of noradrenaline-pre-contracted arteries that do not express functional constrictor a_2_-adrenoceptors (Artigues-Varin et al., 2002; Hansen et al., 2019).

To study the purinergic component of the sympathetic response, desensitization of P2X1-receptors with α,β-methylene ATP that is resistant to hydrolysis by ectonucleotidases (Sjoblom-Widfeldt et al., 1990; Yang and Chiba, 1999a; Tarasova et al., 2003) can be performed. Later, PPADS was introduced as a P2X receptor blocker (Ziganshin et al., 1994; Park et al., 2007). In recent studies, the P2X1-specific purinergic receptor antagonist NF449 is often used to inhibit the purinergic component (Mittal et al., 2020; Gonzalez-Montelongo and Fountain, 2021).

The role of NPY in eliciting/potentiating vessel responses to sympathetic stimulation can be addressed with the use of Y1 receptor antagonists, BIBP 3226 (Prieto et al., 2000) or BIBO3304 (Gonzalez-Montelongo and Fountain, 2021). A potential contribution of postjunctional Y2 receptors to these effects can be studied using their specific antagonist BIIE0246 (Gonzalez-Montelongo and Fountain, 2021).

#### 8.2.2 Analysis of the effects of sensory/parasympathetic nerves

Commonly, sensory nerves can be inactivated by treating the vessels *in vitro* with capsaicin, a pungent ingredient in hot peppers. Capsaicin activates transient receptor potential vanilloid type 1 (TRPV1) channels which are a hallmark of C-type sensory nerve fibers and, therefore, it is an excitotoxin for these fibers (Aalkjær et al., 2021). At a sufficiently high concentration (of the order of a few micromoles), capsaicin induces vasorelaxation mainly due to transmitter release from sensory nerves (Hardebo et al., 1989; Wiencke et al., 1994).

During a 1-h incubation of vessels with capsaicin, sensory nerves become depleted of transmitters. To confirm the depletion, the application should be repeated after the incubation: under such conditions, no relaxation should be observed (Hardebo et al., 1989; Wiencke et al., 1994). Vasorelaxatory effects of CGRP can be selectively blocked by its receptor antagonists, CGRP (8–37) fragment (Wiencke et al., 1994; Del Campo et al., 2009) or BIBN4096BS (De Mey et al., 2008).

NO was shown to contribute substantially to the neurogenic vasorelaxation of isolated arteries (Wiencke et al., 1994; Del Campo et al., 2009). Its effects can be eliminated with the use of NO-synthase inhibitors, such as L-N^ω^-nitro-L-arginine (L-NOARG) (Wiencke et al., 1994) or N^ω^-nitro-L-arginine methyl ester (L-NAME) (Del Campo et al., 2009). In some arteries, such as the bovine ciliary artery, NO-ergic neurogenic vasorelaxation persisted after treatment with capsaicin and endothelium removal but was blocked by TTX pointing to the role of NO as a parasympathetic neurotransmitter (Wiencke et al., 1994).

Notably, NO is a key vasodilator neurotransmitter in the parasympathetic control of vascular tone, with the other transmitters, including acetylcholine, acting as presynaptic modulators of NO release (Roloff et al., 2016; Xavier, 2020). In addition, acetylcholine can exert organ-specific effects on noradrenaline release from sympathetic nerves: reduce it through M2 receptors in peripheral arteries (Simonsen et al., 2002; Hansen et al., 2019) but increase it through α7 nicotinic receptors in cerebral arteries (Si and Lee, 2002). VIP may also participate in the parasympathetic control of cerebral arteries, as seen from the reduced neurogenic relaxation of rabbit basilar arteries in the presence of the VIP receptor antagonist VIP(7–28), but its contribution is rather small (Olivar et al., 2000).

Importantly, the effective concentrations of all pharmacological tools affecting sympathetic, sensory, and parasympathetic intramural nerves may vary in arteries and veins from different species as well as in vessels from different organs. To get more information on this topic, we refer readers to the above-cited articles, as well as to the following reviews (Burnstock and Ralevic, 2014; Xavier, 2020; Aalkjær et al., 2021).

Pharmacological analysis of activated nerve fiber types and their postjunctional effects:

• neurotransmitter secretion from sympathetic and sensory nerve fibers can be disrupted using 6-OHDA/guanethidine and capsaicin, respectively
• postjunctional effects of the neurotransmitters can be blocked with the use of appropriate receptor antagonists; the blockade must be confirmed by applying respective agonists
• at high or even moderate concentrations receptor antagonists may have undesirable side effects

### 8.3 Experimental protocols and data analysis

When vascular responses to EFS of intramural nerves are studied by wire myography, the common procedures of normalization and activation of the preparation are performed at the beginning of each experiment. Notably, high-potassium physiological salt solution should not be used for activation of vascular smooth muscle since it also depolarizes nerve fibers and can deplete them of neurotransmitters. This is especially important in relation to peptide transmitters, whose reserves cannot be replenished by synthesis in varicosities of nerve fibers. For activation of the contractile mechanisms, vessel segments can be exposed several times to the maximal concentration of noradrenaline separated by washout intervals long enough for relaxation (Nilsson, 1984; Sjoblom-Widfeldt et al., 1990; Tarasova et al., 2003). To activate the prejunctional mechanisms, electrical field nerve stimulation is performed.

**FIGURE 12:**
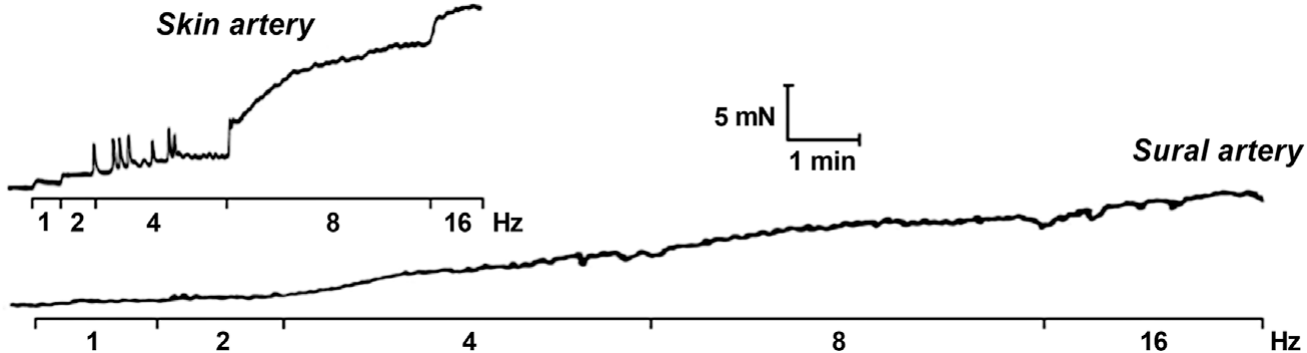
Cumulative frequency–response relationships obtained for segments cut from the medial tarsal branch of the saphenous artery (small skin artery, normalized diameter 225 µm) and from the distal (intramuscular) section of the external sural artery (small skeletal muscle artery, normalized diameter 210 µm).

The general characteristics of responses to sympathetic nerve stimulation can be obtained from cumulative frequency–response relationships (say, in the range of 1–16 Hz), maintaining each frequency until a stable tension level is reached (Nilsson, 1984; Sjoblom-Widfeldt et al., 1990; Tarasova et al., 2003; De Mey et al., 2008). Notably, the time needed to obtain the full frequency–response relationship can vary depending on the arterial type (Figure 12). For example, this time is less than 3 min for mesenteric small arteries, 5–10 min for skin and renal small arteries and 10–15 min for skeletal muscle small arteries (Nilsson, 1984; Tarasova et al., 2003). For sensory nerves frequency–response relationships are performed in a non-cumulative manner (Wiencke et al., 1994), to avoid the depletion of peptide neurotransmitters during long stimulation.

To compare the magnitude of neurogenic contraction of different vessels, responses to nerve stimulation are expressed as a percentage of the maximum contraction in response to noradrenaline. The sensitivity of vessel segments to EFS can be assessed using an EF_50_ value, which is the frequency corresponding to the half-maximum response. The magnitude of neurogenic vasorelaxation can be expressed as a percentage of the pre-contraction level (for more detail see the section “Study of the mechanism of action of contractile or relaxing agonists in isometric vessel preparations”).

A non-cumulative stimulation protocol is used in experiments with antagonists of neurotransmitter receptors (Sjoblom-Widfeldt et al., 1990; Tarasova et al., 2003; Del Campo et al., 2009; Hansen et al., 2019; Mittal et al., 2020). The time between consecutive simulations should be long enough to recover basal tone. Two sets of stimulations with graded frequencies (in the absence and in the presence of receptor antagonists) are performed in the same arterial segment. Time-control experiments should be conducted in parallel in which the second set of stimulations is performed in the presence of the solvent used for the receptor antagonist (See also the section “Design of experimental protocols”).

During data analysis, the effects of the antagonist on the magnitude of neurogenic contractions are evaluated. Importantly, the contribution of the investigated transmitter to the neurogenic response often depends on the frequency of stimulation (Sjoblom-Widfeldt et al., 1990; Wiencke et al., 1994; Tarasova et al., 2003; Del Campo et al., 2009). In addition, the analysis of time characteristics of the response can be performed. For example, the contribution of ATP is relatively high at the beginning of the neurogenic contraction, while noradrenaline mediates the delayed phase of the response (Sjöblom-Widfeldt, 1990; Yang and Chiba, 1999a; Tarasova et al., 2003). Under isobaric conditions, the contribution of ATP to sympathetic neurovascular transmission increases with the rise in transmural pressure to a physiologically relevant level (Rummery et al., 2007).

Protocol features in studies of neurotransmission in isolated vessels:

• do not use high-K^+^ solution during start-up activation of the preparation
• when studying the frequency–response relationship, take into account that full response time at each frequency can vary depending on the arterial type
• do not use long stimulation when studying the effects of sensory nerves, to avoid depletion of non-recovering peptide neurotransmitters
• time control experiments are necessary in experiments with neurotransmitter receptor antagonists
• the contribution of a particular transmitter to the neurogenic response can depend on the frequency and duration of the stimulation as well as on vessel wall stretch

### 8.4 Studying the prejunctional mechanisms of vascular neurotransmission

Excitation-secretion coupling in vascular nerves is regulated by a number of prejunctional receptors and local non-receptor mechanisms (Shepherd and Vanhoutte, 1981; Aalkjær et al., 2021). A simple approach to identify the presynaptic influence of a certain factor is comparing its effects on vasomotor responses to nerve stimulation and to an exogenous transmitter (Nilsson et al., 1985; Martínez et al., 2009). However, this is an indirect approach, not without pitfalls. Next, we will consider two techniques that directly address the presynaptic mechanisms in vascular sympathetic nerves.

#### 8.4.1 Electrochemical detection of noradrenaline release from sympathetic nerves

The method of the electrochemical measurement of the concentration of noradrenaline in the vessel wall is based on the ability of noradrenaline to oxidize upon contact with a positively charged electrode. The recorded oxidation current amplitude is linearly related to the noradrenaline concentration (Gonon et al., 1993; Dunn et al., 1999). To record the oxidation current, a carbon fiber electrode with a diameter of 7–30 μm is gently brought into contact with the vessel wall and a reference Ag/AgCl electrode is placed into the solution in the recording chamber. To improve response sensitivity, carbon fiber electrodes can be either treated electrochemically (Mermet et al., 1990) or coated with a Nafion polymer (Brock and Tan, 2004; Hansen et al., 2019). Although Nafion coatings can slightly slow down oxidation current kinetics, it increases electrode selectivity for catecholamines and protects it from contamination by biomolecules.

At the beginning of such studies, the measurements were performed with the use of differential pulse voltammetry (to determine the noradrenaline oxidation potential) and differential pulse amperometry (to measure the oxidation current at a potential fixed at a pre-determined level) (Mermet et al., 1990; Gonon et al., 1993). It was shown that the noradrenaline oxidation current had a clear peak at +120 mV (Mermet et al., 1990; Gonon et al., 1993). In later studies, a technically simpler method of continuous amperometry was used. Here the electrode potential is maintained at a constant level of 0.3–0.4 V (Mermet et al., 1990; Dunn et al., 1999; Brock and Tan, 2004).

In most studies, the measurements of noradrenaline release were carried out in vessel segments pinned to the elastomer-covered base of the recording chamber, where the vascular wall is not distended properly (Mermet et al., 1990; Gonon et al., 1993; Dunn et al., 1999; Brock and Tan, 2004). Importantly, vessel wall stretch was shown to reduce the distance between sympathetic fibers and SMCs in small arteries (Luff et al., 1987), which could affect noradrenaline kinetics in the neuroeffector junction. In addition, stretch would affect the geometry of intramural nerve plexus and, therefore, could affect the communication between the same type and/or different types of nerve fibers through prejunctional receptors. Thus, the data on noradrenaline release and its prejunctional control obtained under conditions of controlled vascular wall stretch seem to be more physiologically relevant.

**FIGURE 13:**
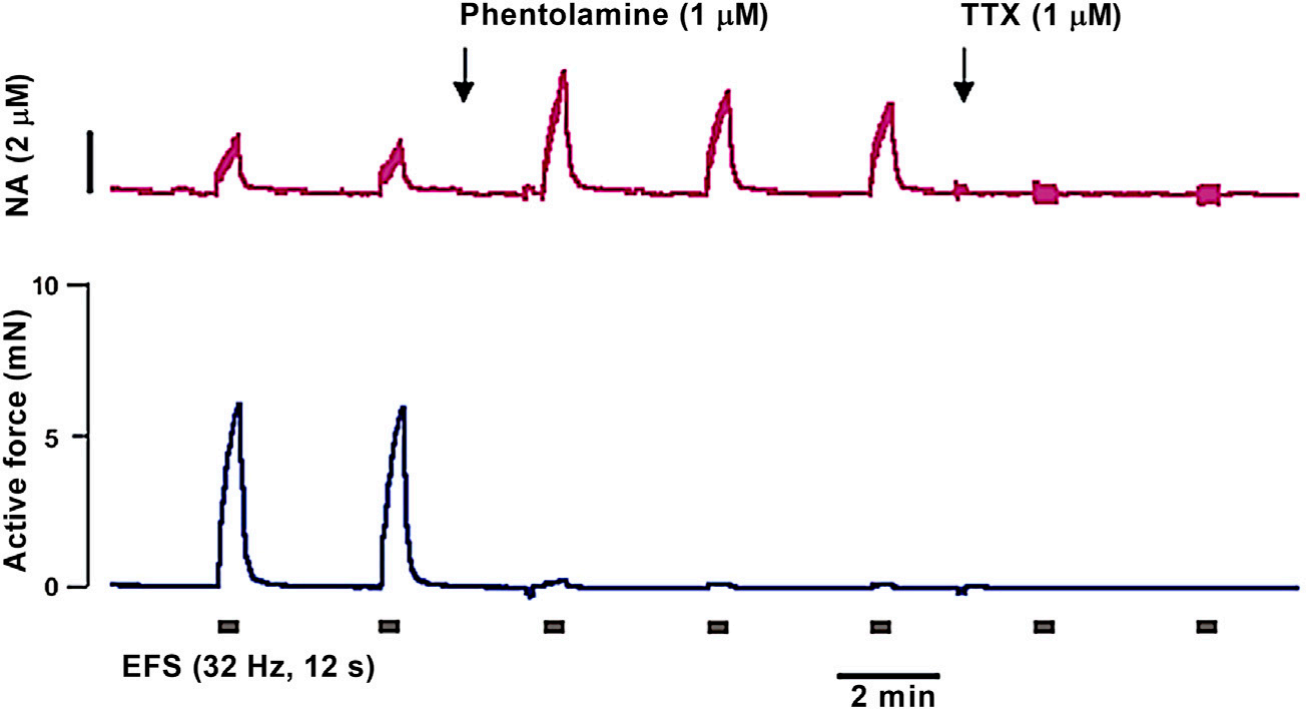
Representative recordings of the noradrenaline concentration (A) and active force (B) in response to EFS of sympathetic nerves by rectangular current pulses (85 mA, 0.1 ms) in an experiment on a segment of the rat mesenteric artery (normalized diameter 280 µm). Phentolamine greatly reduces contraction due to the blockade of postjunctional α_1_-adrenoceptors but increases the concentration of noradrenaline (NA) due to the blockade of prejunctional α_2_-adrenoceptors. In the presence of tetrodotoxin (TTX) contraction and oxidation current completely disappear.

Of note, in wire myographs of conventional design it is difficult to position the electrode so that it is leaning against the vessel wall along a sufficiently long section. However, this can be realized in a wire myograph specially designed for confocal studies and equipped with plastic mounting heads with electrodes (360CW, Danish Myo Technology A/S, Denmark). After mounting the segment, the myograph heads can be rotated to place the segment vertically. Thereafter a carbon fiber electrode is placed alongside the segment in a gently touching position (Hansen et al., 2019). Importantly, simultaneous recording of noradrenaline release and force of contraction makes it possible to distinguish prejunctional and postjunctional effects of various factors (Figure 13).

When conducting a pharmacological analysis of the prejunctional mechanisms by the amperometry technique, it must be considered that some substances can affect the oxidation current of noradrenaline (Dong et al., 2009). It was shown that exposure of carbon fiber electrodes to capsaicin, prazosin, and yohimbine reduced the oxidation current. Therefore, any pharmacological agent used in amperometry experiments should be tested regarding its potential influence on electrode sensitivity and stability.

#### 8.4.2 Studying Ca^2+^ in sympathetic nerves: A picture is worth a thousand words

An increase in the intracellular concentration of free Ca^2+^ ([Ca^2+^]_i_) is a key signal for neurotransmitter secretion (Aalkjær et al., 2021) but the mechanisms of [Ca^2+^]_i_ control in vasomotor sympathetic fibers have been little studied. For small arteries, such studies can be performed by fluorescence confocal microscopy, while the arterial segment is mounted in the confocal model of the wire myograph. Selective loading of the fluorophore into sympathetic fibers is carried out by orthograde axonal transport (Brain and Bennett, 1997). A dextran-linked form of the Oregon Green 488 BAPTA-1 fluorophore with a high quantum yield is used. Dextran (10,000 Da) is a hydrophilic polysaccharide resistant to the action of intracellular glycosidases. It serves as a carrier for the fluorophore along the axons and retains the dye intracellularly.

In experiments on small arteries (Hansen et al., 2019), the proximal end of the arterial segment was gently drawn into a suction pipette, where a drop of a saturated solution of Oregon Green 488 BAPTA-1 is then added. After 4–5 h, the segment is removed from the pipette, the extracellularly adsorbed fluorophore is washed off (for 1–2 h) and the vessel is mounted in the wire myograph. Arterial contractile responses to noradrenaline and EFS should be recorded at the beginning of the experiment, to confirm the functionality of smooth muscle and nerve fibers. The subsequent experiment is conducted in the presence of antagonists of postjunctional receptors, to suppress contraction and displacement of the vessel wall. Excitation is performed at 488 nm and emission light is collected at 515–555 nm. A sampling rate of at least 4 images per second provides enough time resolution for [Ca^2+^]_i_ recordings (Hansen et al., 2019).

During data analysis, the regions of interest (ROIs) corresponding to individual varicosities are selected in the image obtained before the start of nerve stimulation (Brain and Bennett, 1997; Hansen et al., 2019). Then similar ROIs are defined in every image obtained during nerve stimulation; any movement of the varicosity during the stimulation is corrected by an automated procedure (Brain and Bennett, 1997; Hansen et al., 2019). The data on [Ca^2+^]_i_ can be supplemented by force and noradrenaline release data obtained in parallel experiments, which provides a complete picture of sympathetic neurotransmission in small arteries (Hansen et al., 2019).

Studying the prejunctional mechanisms of vascular neurotransmission:

• electrochemical detection of noradrenaline release is best done in myograph-mounted preparations, since noradrenaline kinetics in the neuroeffector junction is potentially dependent on vessel wall stretch
• pharmacological agents can affect sensitivity and stability of the electrode during amperometry measurements
• a fluorophore can be selectively loaded into sympathetic fibers by orthograde axonal transport in a dextran-linked form
• when studying [Ca^2+^]_i_ in sympathetic varicosities, the mechanical response of the vessel is blocked by neurotransmitter receptor antagonists and any residual displacement is corrected by processing using a special software
